# FUS-induced circRHOBTB3 facilitates cell proliferation via miR-600/NACC1 mediated autophagy response in pancreatic ductal adenocarcinoma

**DOI:** 10.1186/s13046-021-02063-w

**Published:** 2021-08-20

**Authors:** Taoyue Yang, Peng Shen, Qun Chen, Pengfei Wu, Hao Yuan, Wanli Ge, Lingdong Meng, Xumin Huang, Yuzhe Fu, Yihan Zhang, Weikang Hu, Yi Miao, Zipeng Lu, Kuirong Jiang

**Affiliations:** 1 Pancreas Center, the First Affiliated Hospital of Nanjing Medical University, Nanjing, China; 2 Pancreas Institute, Nanjing Medical University, Nanjing, China; 3 Nanjing Medical University, Nanjing, China

**Keywords:** CircRNAs, PDAC, miR-600, NACC1, Autophagy

## Abstract

**Background:**

Circular RNAs (circRNAs) are becoming a unique member of non-coding RNAs (ncRNAs) with emerging evidence of their regulatory roles in various cancers. However, with regards to pancreatic ductal adenocarcinoma (PDAC), circRNAs biological functions remain largely unknown and worth investigation for potential therapeutic innovation.

**Methods:**

In our previous study, next-generation sequencing was used to identify differentially expressed circRNAs in 3 pairs of PDAC and adjacent normal tissues. Further validation of circRHOBTB3 expression in PDAC tissues and cell lines and gain-and-loss function experiments verified the oncogenic role of circRHOBTB3. The mechanism of circRHOBTB3 regulatory role was validated by pull-down assays, RIP, luciferase reporter assays. The autophagy response of PANC-1 and MiaPaca-2 cells were detected by mCherry-GFP-LC3B labeling and confocal microscopy, transmission electron microscopy and protein levels of LC3B or p62 via Western blot.

**Results:**

circRHOBTB3 is highly expressed in PDAC cell lines and tissues, which also promotes PDAC autophagy and then progression in vitro and in vivo. Mechanistically, circRHOBTB3 directly binds to miR-600 and subsequently acts as a miRNA-sponge to maintain the expression level of miR-600-targeted gene NACC1, which facilitates the autophagy response of PDAC cells for adaptation of proliferation via Akt/mTOR pathway. Moreover, the RNA-binding protein FUS (FUS) directly binds to pre-RHOBTB3 mRNA to mediate the biogenesis of circRHOBTB3. Clinically, circRHOBTB3, miR-600 and NACC1 expression levels are correlated with the prognosis of PDAC patients and serve as independent risk factors for PDAC patients.

**Conclusions:**

FUS-mediated circRHOBTB3 functions as a tumor activator to promote PDAC cell proliferation by modulating miR-600/NACC1/Akt/mTOR axis regulated autophagy.

**Supplementary Information:**

The online version contains supplementary material available at 10.1186/s13046-021-02063-w.

## Background

Pancreatic ductal adenocarcinoma (PDAC) has emerged as one of the most lethal cancer types, killing on an annually basis more than 48,220 patients in the United States. The 5-year survival rate across all stages of PDAC is 10%, with slowly improvement falling behind most other neoplastic diseases [1]. Medical management of PDAC is challenging as around 15 to 20% of patients are diagnosed at a resectable tumor stage, while other therapies are dominated by chemotherapy or adjuvant chemotherapy [2]. There are encouraging news that effective combination chemotherapeutic regimens have prolong the survival of patients with metastatic pancreatic ductal adenocarcinoma (PDAC), such as mFOLFIRINOX (fluorouracil, leucovorin, irinotecan, and oxaliplatin) and gemcitabine hydrochloride plus nanoparticle albumin-bound paclitaxel (GA) [3]. However, patient survival remains disappointing, urging us to step up developing biomarker-selected therapy in PDAC.

Circular RNAs (circRNAs) are endogenous biomolecules in eukaryotes with tissue-specific and cell-specific expression patterns, characterized by a continuous covalent closed loop without a 5′-cap structure or 3′-poly A tail [4]. Its biogenesis is formed by an uncanonical linkage termed ‘back-splice’ between a downstream 3′ splice site and an upstream 5′ splice site in a linear pre-messenger RNA [5]. The back-splicing relies on looping structure of the intron flanking sequences on each side which brings the donor site and the acceptor site approximate to each other [6]. This looping can be mediated by base pairing between inverted repeat elements (such as Alu elements), or by the combination of RNA-binding proteins (RBPs) (such as Protein quaking (QKI) or RNA-binding protein FUS (FUS) and the specific motifs in the flanking introns [7–9]. There is mounting evidence that circRNAs exist in various malignant cells and regulate a broad range of biological processes, such as tumor formation, progression, relapse, and drug resistance [10]. Diverse functional characteristics of circRNAs are constantly emerging, including serving as miRNA sponge, interacting with proteins, regulating transcription and splicing, and translated into peptides [11, 12]. The underlying mechanisms of circRNAs in the pathogenesis of PDAC remain largely unclear and need further exploration and may provide new sight to the targeted therapy of PDAC due to its distinctive structure and rich functionality.

Nucleus accumbens-1 (NAC1), encoded by the NACC1 gene, functions as a transcription factor repressor that belongs to the bric-a-brac Tramtrack Broad complex/pox virus and Zn finger (BTB/POZ) family [13]. There are emerging studies finding NAC1 overexpressed in several types of human carcinomas including ovarian cancer, cervical cancer, breast cancer, and colon cancer [14]. It participates in various tumor biological processes such as cell growth and survival, migration, and invasion, and resistance to chemotherapeutic drugs [15]. One of the molecular mechanisms underlying the essential role of NAC1 in cancer cell survival are recently reported to involve in autophagic response mediated by high-mobility group protein B1 (HMGB-1) [16]. The relentless advances in the understanding of autophagy have always given rise to debate about whether it is tumor promoting or inhibiting [17]. Despite this potential for confusion, clinical trials have been carried out to intervene autophagy in cancer therapy, mostly focused on inhibiting autophagy [18]. In PDAC, a combination of autophagy inhibition and immune checkpoint blockade (ICB) therapy could become reality with theoretical basis by which enhanced autophagy degrades MHC-1 selectively to facilitate immune evasion [19]. However, the regulatory relationship between NAC-1 and autophagy, and the effect of circRNAs on it remain largely unexplored.

In this study, we identified a novel autophagy promotive circRNA circRHOBTB3, induced by FUS, which is highly expressed in PDAC. Our data further demonstrated that circRHOBTB3 acts as a miRNA-sponge to maintain the expression level of miR-600-targeted gene NACC1, thereby increasing autophagic flux of PDAC cells for adaptation of tumor development through inhibiting Akt/mTOR pathway. These findings extend the understanding of circRNA and autophagy in PDAC progression and highlight the significance of circRHOBTB3 in the biomarker-selected or combination therapy.

## Methods

### Patients and tissue specimens

Tumor tissues and adjacent normal pancreas tissues were collected from PDAC patients who received pancreaticoduodenectomy at Pancreas Center, the First Affiliated Hospital of Nanjing Medical University, from Jul. 2014 to Dec. 2018. The 110 patients were followed regularly until 11th September 2020. All patients didn’t receive any chemotherapy or radiotherapy before surgery. All patients signed an informed consent that was supervised by the Hospital Ethics Committee before specimen collection. None of the 110 selected patients died within 1 month after surgery. The samples were excised from patients, immediately frozen in liquid nitrogen and stored until use. All the cancer and adjacent tissues were diagnosed by two pathologists independently. TNM stage classification complied with the TNM classification system of the International Union Against Cancer (8th edition). We used Kaplan Meier method to draw the overall survival curve according to the relative expression of circRHOBTB3 (or miR-600 or NACC1) and the cut-off value (Median of the expression) for circRHOBTB3 (or miR-600 or NACC1).

### Cell culture and transfection

Human PDAC cell lines (BxPC-3, MiaPaca-2, CFPAC-1 and PANC-1) and human pancreatic ductal epithelial (HPNE) cells were purchased from the Cell Bank of Type Culture Collection of the Chinese Academy of Sciences in Shanghai, China. The cells were cultured in a humidified atmosphere at 37 °C with 5% CO2. All cells were cultured in Dulbecco’s modified Eagle’s medium (Life Technologies) with 10% fetal bovine serum (Wisent, Montreal, QC, Canada), 10 mM HEPES (Sigma, St Louis, MO), 2 mM L-glutamine (Sigma), 1 mM sodium pyruvate (Sigma), 100 U/ml of penicillin (Life Technologies) and 100 μg/ml streptomycin (Life Technologies).

Plasmids were transfected in to PDAC cells using Lipofectamine 3000 and P3000 (Invitrogen) according to the manufacturer’s instructions. SiRNAs, miRNA mimics or inhibitors were transfected into cells using Lipofectamine 3000 (Invitrogen, USA). To construct the circRHOBTB3 stably knocking down cell lines, we transfected the control vector or the circRHOBTB3 knocking down vector pLV3ltr-GFP-Puro-U6-siRNA (circRHOBTB3) into PANC-1 and MiaPaca-2 cells and then selected them with puromycin (Sigma, USA) for 2–3 weeks until circRHOBTB3 was stably knocked down in the cells.

### SiRNAs, miRNA and plasmid construction

The circRHOBTB3 overexpression vector was constructed by Obio Technology Corp., Ltd. (Shanghai, China) by inserting the sequence of human circRHOBTB3 cDNA into the pGL3-circ expression vector, with the empty plasmid used as a control. siRNA targeting circRHOBTB3 and the control siRNA were synthesized by RiboBio (Guangzhou, China).

The miR-600 mimics and inhibitor, NACC1-overexpressing vector and NACC1 siRNAs were synthesized by GenePharma (Shanghai, China). The pRL-SV40-circRHOBTB3 and NACC1 luciferase reporter was constructed by inserting circRHOBTB3 fragments and 3’UTR fragment of NACC1 downstream of the luciferase reporter gene in the reporter plasmid (GenePharma, Shanghai, China). The miR-600 complementary sequence “CTGTAAG” in circRHOBTB3 and the 3′UTRs of NACC1 were mutated to remove the complementarity. All of the constructs were verified by sequencing and the sequences are listed in Table S1.

### RNA extraction and RT-qPCR

Total RNA was isolated from clinical specimens or cell lines with TRIzol Reagent (Life Technologies, Carlsbad, CA, USA) according to the manufacturer’s instructions. After spectrophotometric quantification, RNA (500 ng) was reverse transcribed into cDNA following the protocol of the iScript cDNA Synthesis Kit (Bio-Rad, Hercules, CA, USA).

The circRNA and mRNA levels were normalized to that of 18S rRNA or GAPDH in tissue or cell lines, respectively. The miRNA was normalized to that of U6. Target gene expression was calculated using the 2^-ΔΔCT^ method. Each quantitative PCR assay was performed in triplicate and independently repeated three times. The sequences of the primers used in the present study are listed in Table S1.

### RNase R and Actinomycin D treatment

For RNase R treatment, 2 μg of total RNA was incubated for 10 min at 37 °C with or without 3 U/μg RNase R (Epicentre Technologies, Madison, WI, USA). PDAC cells were treated with 5 μg/ml actinomycin D (Sigma-Aldrich, USA) and collected in a series of time intervals. The expression of circRHOBTB3 and the linear mRNA was detected by qRT-PCR.

### Isolation of nuclear and cytoplasmic fractions

Cytoplasmic and nuclear fractions were preparing using the reagents in a PARIS™ kit (AM1556, Thermo Fisher Scientific, Waltham, USA) according to the manufacturer’s protocol. Briefly, PDAC cells were lysed in Cell Fraction Buffer on ice for 10 min. After centrifugation at 500 g for 3 min at 4 °C, the supernatant was collected as the cytoplasmic fraction. Then, the pelleted nuclei were washed with Cell Fraction Buffer and used as the nuclear fraction.

### Fluorescence in situ hybridization (FISH)

Cy3-labelled circRHOBTB3 probes and fluorescein amidite (FAM)-labelled miR-600 probes were designed and synthesized by RiboBio. The sequences of the probes are listed in Table S1. A fluorescence in situ hybridization (FISH) kit (RiboBio) was used to detect the probe signals in PDAC cells and tissues according to the manufacturer’s instructions. Nuclei were stained with 4,6-diamidino-2-phenylindole (DAPI). All images were acquired with an LSM880 NLO (2 + 1 with BIG) confocal microscope system (Carl Zeiss).

### Cell proliferation assay

We used a CCK-8 assay kit (Dojindo, Japan), clone formation assay and 5-Ethynyl-20- deoxyuridine (EdU) assay (Beyotime) to assess cell proliferation. The CCK-8 assay was performed by seeding the treated cells in 96-well plates at 1.0 × 10^3^ cells/well. Subsequently, each well was incubated with 100ul 10% CCK-8 solution for 2 h at 37 °C away from light and the absorbance of each well at 450 nm was measured with a microplate reader. For clone formation assay, cells were seeded in six-well plates (800 cells/well) and cultured in complete medium supplemented with 10% fetal bovine serum for 2 weeks. Then, the clusters were stained with 0.1% crystal violet (Beyotime) counted if their diameter was greater than 1 mm. The EdU assay was performed according to the manufacturer’s protocol. Briefly, PDAC cells were plated in 96-well plates and incubated with a 50 mM EdU solution for 2 h and then fixed in 4% paraformaldehyde. The cells were then permeabilized with 0.3% Triton for 10 min and then sequentially stained with Alexa Fluor 555 azide and Hoechst 33342. Subsequently, the EdU-treated cells were imaged and counted under an Olympus FSX100 microscope (Olympus, Tokyo, Japan).

### RNA pull-down assay

A total of 1.0 × 10^7^ cells were harvested and lysed, and C-1 magnetic beads were incubated with the circRHOBTB3 probes or miR-600 probes (Life Technologies) at 25 °C for 2 h to generate probe-coated beads. The cell lysates were then incubated with the coated beads at 4 °C overnight. The RNA complexes bound to the beads were then eluted and extracted with a RNeasy Mini Kit (Qiagen) for qRT-PCR analysis. The biotinylated probes were designed and synthesized by RiboBio (Guangzhou, China) and the sequences of the probes are listed in Table S1.

### RNA immunoprecipitation (RIP) assay

The Magna RIP RNA-Binding Protein Immunoprecipitation Kit (Millipore, Billerica, MA, USA) was used to perform the RIP assay. PDAC cells were collected and lysed in RIP lysis buffer supplemented with protease and RNase inhibitors. The cell lysates were then incubated with IgG, anti-AGO2 or anti-FUS antibody-coated beads (Millipore) at 4 °C overnight. The immunoprecipitated RNAs were subsequently extracted with a RNeasy MinElute Cleanup Kit (Qiagen, Valencia, CA, USA) after treatment with proteinase K buffer. Finally, the RNA levels of the assayed genes were measured by qRT-PCR.

### Western blot

Proteins were extracted from treated PDAC cells with RIPA buffer containing proteinase inhibitor. The protein concentrations in the cell lysates were measured by the DC Protein Assay Kit (Bio-Rad). Then, the proteins were separated via electrophoresis using SDS-containing polyacrylamide gels and then transferred onto a polyvinylidene fluoride (PVDF) membranes (Millipore, Billerica, MA, USA). After blocking the membranes with 5% nonfat dry milk in 0.1% Tween (TBST) buffer at room temperature for 2 h, the membranes were incubated at 4 °C overnight with the appropriate primary antibody. Subsequently, the membranes were washed 3 times with TBST buffer, and the membranes were incubated with a corresponding HRP-labelled secondary antibody for 2 h at room temperature, after which they were washed 3 times with TBST buffer. Finally, the western blot signals were visualized using an enhanced chemiluminescence detection system with Chemiluminescence HRP Substrate (Millipore, WBKL0100). All the primary and secondary antibodies used in this study are listed in Table S2.

### Mouse xenograft model

Four-week-old male nude mice (BALB/c) were purchased from the Animal Center of Nanjing Medical University (Nanjing, China). All animal experiments were conducted in compliance with animal protocols approved by Nanjing Medical University, and were carried out at the Animal Center of Nanjing Medical University. Six mice per group were subcutaneously injected in the inguinal region with 1 × 10^6^ circRHOBTB3 lentivirus treated cells. The tumor volume was measured every 5 days and calculated according to the following formula: volume = (width^2^ × length)/2. After 25 days, all the mice were sacrificed, and then tumors were resected and collected. All experiments were performed following the relevant institutional and national guidelines and regulations.

### Immunohistochemistry (IHC)

Human tumor tissues and xenografts were first fixed in 4% paraformaldehyde and embedded in paraffin, and 5 μm thick sections were cut. Then, antigen retrieval was performed by incubating the samples with sodium citrate buffer (pH 6.0) for 20 min at 95 °C, after which the samples were blocked with 5% normal goat serum for 10 min at 20 °C. Subsequently, the sections were incubated with polyclonal antibodies against NAC1 or Ki-67 at 4 °C overnight and then incubated with secondary antibodies. The tissue sections were scanned, and the protein levels were calculated as positive cells/total cells by Halo v3.0.311.314. All the primary and secondary antibodies used in this section are listed in Table S2.

### Autophagy flux detection in cells

PANC-1 and MiaPaca-2 cells transfected with mCherry-GFP-LC3 lentivirus (GeneChem, China) were seeded into a 35-mm culture dish for confocal microscopy. The nucleus was stained with DAPI. Red and yellow puncta representing autolysosomes and autophagosomes, respectively, were detected by confocal microscopy (Carl Zeiss, Germany). At least 10 cells in each of three independent experiments were analyzed randomly.

### Transmission electron microscopy (TEM)

We placed the cell pellet in a droplet of 2.5% glutaraldehyde in PBS buffer at pH 7.2 and fixed the cells overnight at 4 °C. The samples were then rinsed in PBS solution for 10 min three times and postfixed in 1% osmium tetroxide for 60 min at room temperature. Next, the samples were embedded in 10% gelatin, fixed in glutaraldehyde at 4 °C and cut into several blocks. Subsequently, the samples were dehydrated for 10 min in increasing concentrations of alcohol (30, 50, 70, 90, 95, and 100% × 3). Next, we exchanged alcohol with propylene oxide and infiltrated samples with increasing concentrations (25, 50, 75, and 100%) of Quetol-812 epoxy resin mixed with propylene oxide. Each step lasts at least 3 h. The samples were then embedded in pure Quetol-812 epoxy resin and polymerized at 35 °C for 12 h, 45 °C for 12 h, and 60 °C for 24 h. We cut samples into sections (100 nm) using an ultramicrotome and poststained them with uranyl acetate for 10 min and lead citrate for 5 min at room temperature. After that, we observed sections under a transmission electron microscope operated at 120 kV.

### Statistical analysis

Data are presented as the means ± standard deviations (SDs). The general statistical analysis was performed using GraphPad Prism 8.0 (GraphPad Software, La Jolla, USA). Student’s t-test was used to analyze the differences between two groups, while two-way ANOVA was used for multiple groups. Correlations between circRHOBTB3, miR-600 and NACC1 expression and various clinicopathological or serological variables were analyzed by the Mann-Whitney U test. Survival distributions and overall survival (OS) rates were determined using the Kaplan-Meier method, and the significance of differences between survival rates was calculated by the log-rank test. The univariate and multivariate Cox proportional hazards model, which was used to estimate the adjusted hazard ratios and 95% confidence intervals, as well as identify independent prognostic factors, was performed by SPSS 20.0 (IBM, SPSS, Chicago, IL, USA).

## Results

### Identification of a circRHOBTB3 formed by exon6 and exon7 of RHOBTB3 upon back-splicing

**Fig. 1:**
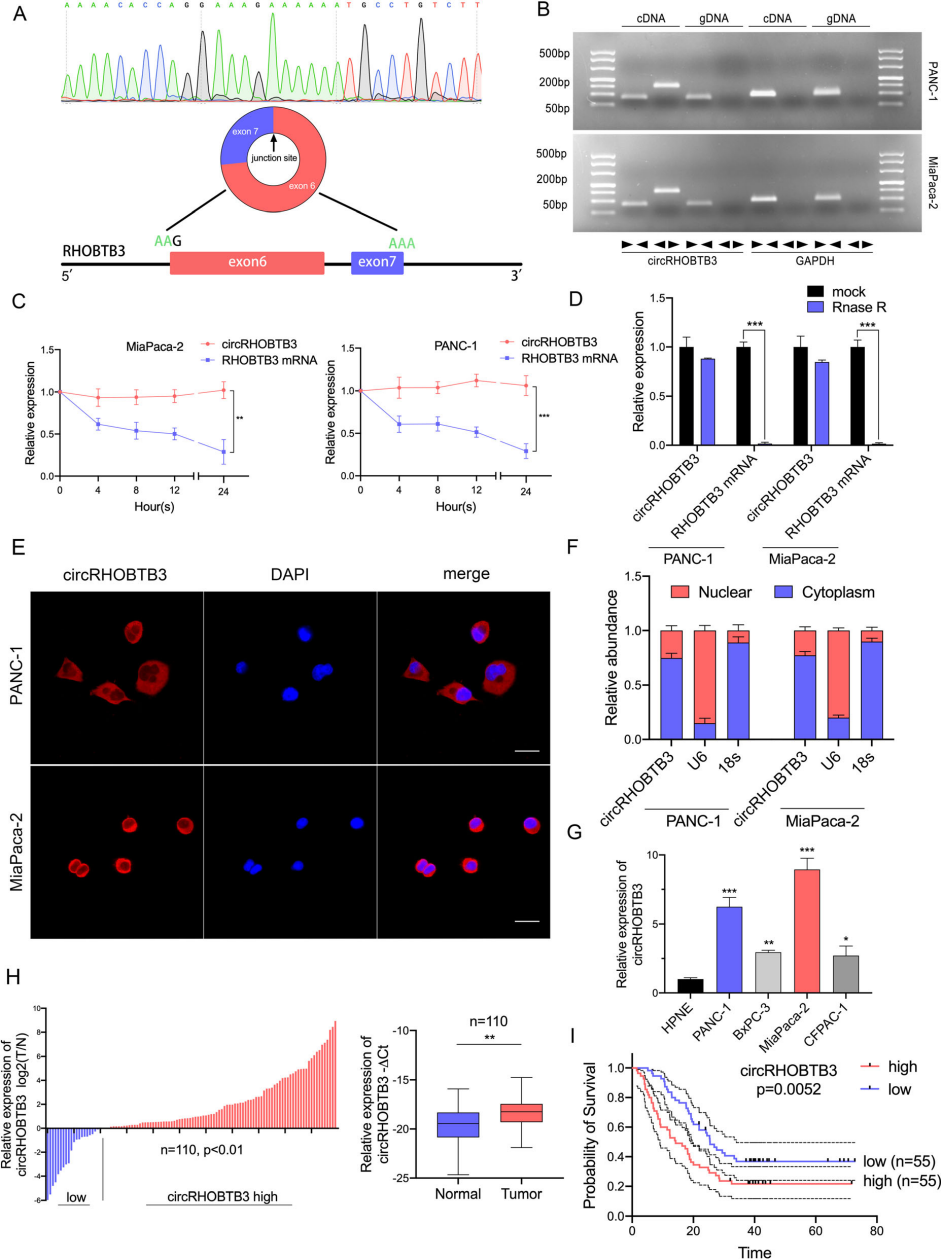
Identification and characteristics of circRHOBTB3 in PDAC cells and tissues. **a** Schematic illustration of the genomic location and back splicing of circRHOBTB3, with the junction site validated by Sanger sequencing. **b** PCR and agarose gel electrophoresis experiments confirmed the circular form of circRHOBTB3 using divergent and convergent primers in PDAC cDNA and gDNA samples. **c** Actinomycin D treatment was applied to evaluate the stability of circRHOBTB3 and RHOBTB3 mRNA in PDAC cells. **d** circRHOBTB3 exhibited higher stability than RHOBTB3 mRNA in PDAC cells after treatment of RNase R. **e** FISH experiments showed that circRHOBTB3 predominantly located in cytoplasm in PANC-1 and MiaPaca-2 cells, the circRHOBTB3 probe was labeled with Cy3(red), while nuclei were stained with DAPI. **f** Nuclear and cytoplasm fractionation assays revealed the subcellular location of circRHOBTB3. **g** Relative circRHOBTB3 expression in various cell lines of PDAC was determined by qRT-PCR. **h** Relative circRHOBTB3 expression in PDAC tissues and adjacent normal tissues was measured by qRT-PCR (*n* = 110). **i** The Kaplan-Meier overall survival curve of PDAC patients with either high or low circRHOBTB3 expression level in tumor. The samples were imaged at 1000× magnification. Scale bar = 10 μm. All data are presented as the means ± SD of three independent experiments. *p < 0.05, **p < 0.01, ***p < 0.001

Our previous studies had characterized circular RNA transcripts using RNA-seq analysis of ribosomal RNA-depleted total RNA from three pairs of pancreatic ductal carcinoma and adjacent normal tissues, and identified circNEIL3 as an oncogene for PDAC progression and metastasis [20]. In this research, we characterized a circRNA derived from RHOBTB3 gene, circRHOBTB3 (chr5:95091099–95,099,324, circBase ID: hsa_circ_0007444). Based on the circBase annotation, circRHOBTB3 is a 479 nt length circRNA derived from exon6 and exon7 of the parental gene through back-splicing. Sanger sequencing confirmed the junction site with the PCR product (Fig. 1a). The agarose gel electrophoresis of the PCR products showed that circRHOBTB3 was only amplified from cDNA by divergent primers, ruling out the possibility of genomic rearrangements and trans-splicing (Fig. 1b). The half-time of the circRHOBTB3 transcript was significantly longer than RHOBTB3 mRNA after treated with actinomycin D, which suppressed RNA transcription. Moreover, the RNase R assays showed that circRHOBTB3 was resistant to RNase R treatment, which is a 3′ to 5′ exoribonuclease, while the linear counterpart mRNA considerably degraded after the enzyme treatment, illustrating the circular form of circRHOBTB3. (Fig. 1c-d). Furthermore, we investigated the cellular localization of circRHOBTB3 in PDAC cell lines, nuclear and cytoplasm fractionation and FISH assays indicated that circRHOBTB3 is mainly located in cytoplasm (Fig. 1e-f). We also detected higher circRHOBTB3 expression in four PDAC cell lines and 110 PDAC samples relative to the adjacent normal tissue samples via qRT-PCR (Fig. 1g-h), and its higher expression was also associated with poor overall survival (OS) than lower groups based on median expression (Fig. 1i). Collectively, these results demonstrated that circRHOBTB3, located in the cytoplasm of PDAC cells, is a highly expressed and stable circRNA.

### CircRHOBTB3 accelerates the proliferation of PDAC cells in vitro and in vivo

**Fig. 2:**
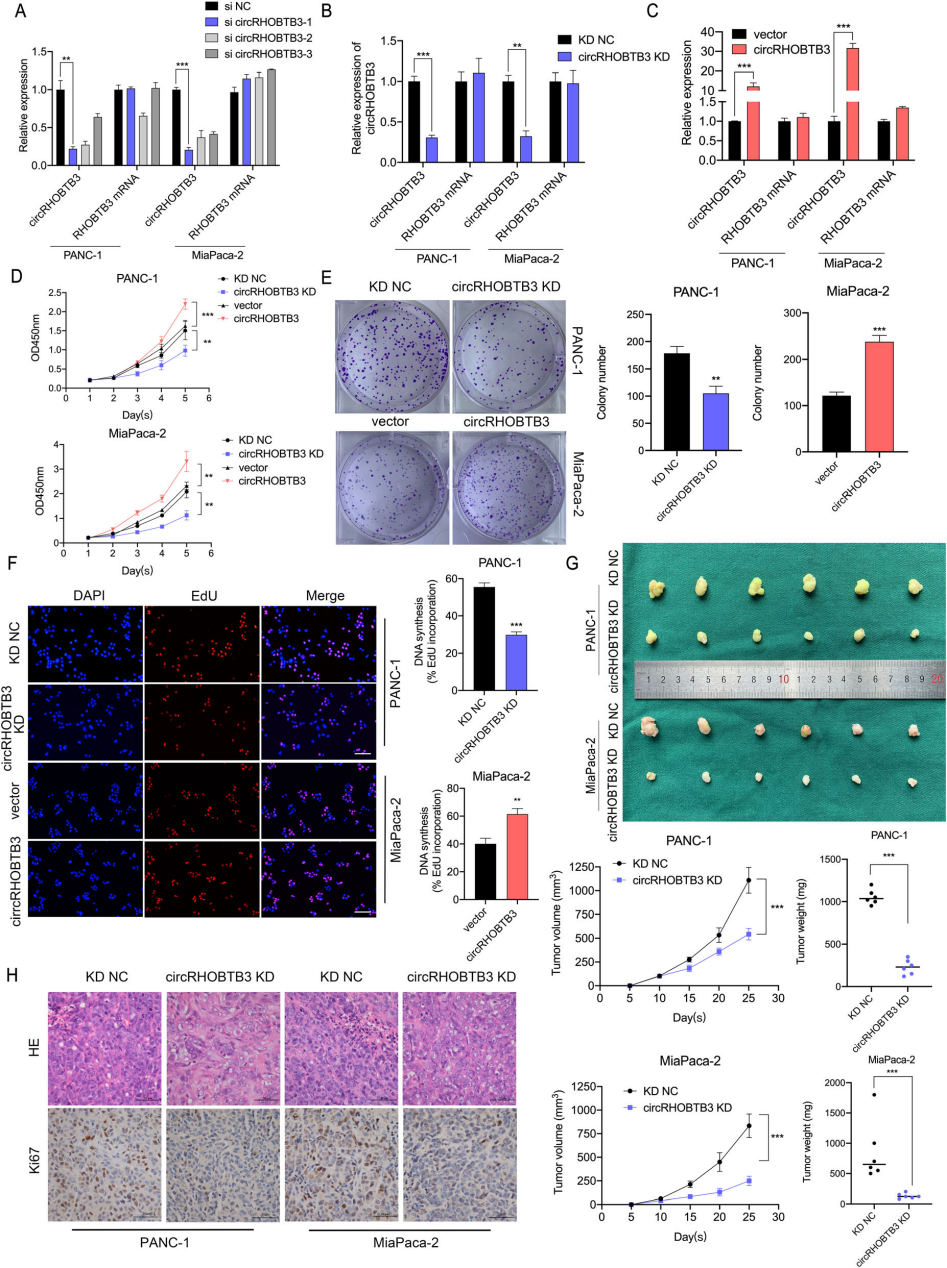
circRHOBTB3 promotes the proliferation of PDAC cells in vitro and in vivo. **a** the efficiency of different siRNAs on cicRHOBTB3 expression level was determined with qRT-PCR in PANC-1 and MiaPaca-2 cell lines. **b-c** The knocking-down and overexpression efficiency of circRHOBTB3 were detected with qRT-PCR. **d** The growth curves of PANC-1 and MiaPaca-2 cells were evaluated with CCK-8 assays after knocking-down and overexpression of circRHOBTB3. **e** Colony formation assays were performed to evaluate cell proliferation. **f** EdU assays were performed to evaluate cell proliferation abilities. The samples were imaged at 200X magnification. Scale bar = 50 μm. **g** Representative images of subcutaneous xenograft tumors (*n* = 6 for each group). Analysis on tumor weight and volume showed decreased tumor growth after knocking-down of circRHOBTB3. **h** HE and Ki67 staining of xenograft tumors showing inhibited proliferative rate after circRHOBTB3 knockdown. Figures were imaged at 400× magnification. Scale bar = 50 μm. All data are presented as the means ± SD. *p < 0.05, **p < 0.01, ***p < 0.001

To investigate the biological functions of circRHOBTB3 in PDAC cells, three siRNAs that specifically targeted the junction sites of circRHOBTB3 were constructed and transfected into PANC-1 and MiaPaca-2 cells (Fig. 2a). Based on the sequence of si-circRHOBTB3–1, we constructed knockdown lentivirus package to acquire stably transfected cell lines (Fig. 2b). And pGL3-circRHOBTB3 plasmid was used to overexpress circRHOBTB3 (Fig. 2c). Consequently, circRHOBTB3 expression level was significantly downregulated or upregulated in both PANC-1 and MiaPaca-2 cell lines, while the parental gene RHOBTB3 expression was barely affected. Based on these cell lines with different circRHOBTB3 expression level, gain-of-function assays were performed to evaluate the effects of circRHOBTB3 on the malignant potentials of pancreatic cancer cells with CCK8, colony formation and EdU incorporation assays. As shown, knocking-down circRHOBTB3 in pancreatic cells significantly inhibited cellular proliferation and colony formation, while overexpression showed the opposite effects (Fig. 2d-f, Figure S1a-b).

To further investigate the effects of circRHOBTB3 on tumor growth in vivo, mouse models of xenograft tumor growth were performed. We found that the tumor weight and volume were considerably reduced upon circRHOBTB3 knockdown, indicating a vital role of circRHOBTB3 in tumor growth (Fig. 2g). Meanwhile, Ki67 staining of xenograft tumor tissue demonstrated that in PANC-1 and MiaPaca-2 cells, circRHOBTB3 knockdown significantly suppressed proliferative activity in vivo (Fig. 2h). Taking together, these findings demonstrated that circRHOBTB3 plays a promotive role in PDAC proliferation in vivo and vitro.

### CircRHOBTB3 serves as a sponge for miR-600 in PDAC cells

**Fig. 3:**
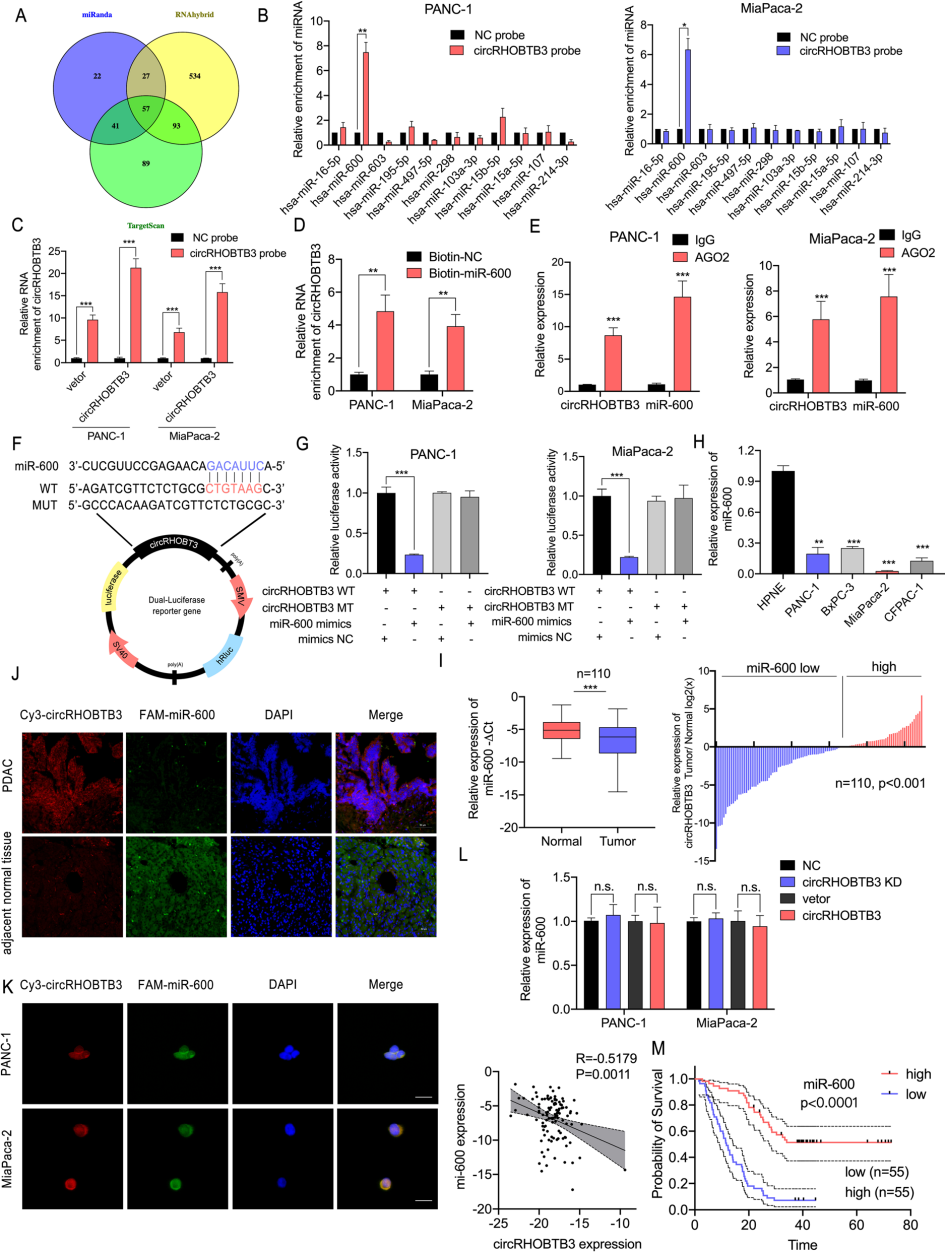
circRHOBTB3 acts as a sponge for miR-600. **a** The Venn diagram revealed the overlap of potential target miRNAs for circRHOBTB3 with prediction by miRanda, RNAhybrid and TargetScan. **b** The efficiency of circRHOBTB3 probe in PANC-1 and MiaPaca-2 cells were validated with qRT-PCR assays. **c** The 11 candidate miRNAs were selected to perform pull-down assays and their enrichment levels were detected with qRT-PCR. **d** Pull-down assays demonstrated the enrichment of circRHOBTB3 using biotin-labeled miR-600 probe. **e** RNA immunoprecipitation by AGO2 antibody showed that both circRHOBTB3 and miR-600 were significantly enriched in PANC-1 and MiaPaca-2 cell lines. **f** A schematic diagram of luciferase reporter plasmid with wild-type (WT) or mutant (MT) circRHOBTB3 sequence and showing its binding site for miRNA-600. **g** The luciferase activities of the circRHOBTB3 luciferase reporter plasmid (WT and MT) were detected in PANC-1 and MiaPaca-2 cells transfected with miR-600 mimics or mimic NC. **h** The relative expression of miR-600 in PDAC tissues and adjacent normal tissues were determined by qRT-PCR. **i** The miR-600 expression was detected in four PDAC cells lines and normal pancreatic epithelial cells (HPNE). **j-k** The FISH assay on both tissues and cell levels validated the colocalization of circRHOBTB3 and miR-600. The circRHOBTB3 probe was labeled with Cy3 (red), miR-600 probes were labeled with FAM (green), and nuclei were stained with DAPI (blue). The samples were imaged at 1000× magnification. Scale bar = 10 μm. **l** The Pearson correlation analysis showed that circRHOBTB3 expression is negatively associated with miR-600 expression in PDAC tissue. The miR-600 expression levels were determined by qRT-PCR upon circRHOBTB3 knockdown or overexpression. **m** The Kaplan-Meier curve showed that lower expression of miR-600 is correlated with shorter overall survival (OS) in PDAC patients (*n* = 110). All data are presented as the means ± SD of three independent experiments. *p < 0.05, **p < 0.01, ***p < 0.001

CircRNAs mainly exhibited their biological functions in tumor progression by sponging to miRNAs in abundant previous studies [21]. To determine the miRNAs interacting with circRHOBTB3, we overlapped the results obtained from three public database miRanda (http://www.microrna.org), RNAhybrid (https://bibiserv.cebitec.uni-bielefeld.de/rnahybrid/) and Targetscan (http://www.targetscan.org/mamm_31/) (Fig. 3a). A total of 57 miRNAs with predicted capacity of binding circRHOBTB3 were suggested and 11 candidate miRNAs with top confidence were selected (Table S3). Then, we performed RNA pull-down assays to further confirm the binding counterpart miRNAs of circRHOBTB3. The efficiency of circRHOBTB3 probe was demonstrated in circRHOBTB3 overexpression and control vector transfected cells (Fig. 3b). Among the 11 selected miRNAs, miR-600 was specifically enriched by circRHOBTB3 biotin-labelled probe in PANC-1 and MiaPaca-2 cell lines (Fig. 3c). On the other hand, an RNA pull-down assay using biotin-labelled miR-600 probe was performed, showing circRHOBTB3 was significantly enriched with biotin-miR-600 probe but not with the control probe in pancreatic cancer cells (Fig. 3d). Moreover, the function of circRNA as miRNA sponge requires binding with AGO2 and miRNA to form a circRNA-AGO2-miRNA complex [22]. Anti-AGO2 RNA immunoprecipitation (RIP) experiments confirmed that both circRHOBTB3 and mi-600 could bind to AGO2, indicating circRHOBTB3 could act as a miR-600 sponge. (Fig. 3e). To confirm the sponge effect of circRHOBTB3, we conducted a dual-luciferase assay by co-transfection of miR-600 mimics and a circRHOBTB3-WT or mutated plasmid with a luciferase reporter in PANC-1 and MiaPaca-2 cells respectively (Fig. 3f). The results showed that the luciferase activity of wild-type reporter was considerably reduced after transfection of miR-600 mimics, whereas no changes was noticed in the mutant reporter, demonstrating that circRHOBTB3 acts as a miR-600 sponge specifically binding at the CTGTAAG sites (Fig. 3g). Next, we checked the miR-600 expression level in different pancreatic cell lines and found that miR-600 expression was relatively lower in PDAC cell lines than HPNE cells (Fig. 3h). We further detected miR-600 expression in 110 pairs of PDAC tissues and adjacent normal tissues and the results showed that PDAC tissues exhibit decreased expression of miR-600 than adjacent normal tissues (Fig. 3i). Furthermore, FISH assays demonstrated that circRHOBTB3 and miR-600 co-localized in cytoplasm in pancreatic cancer cell lines and PDAC tissue (Fig. 3j, k). Besides, miR-600 expression was not influenced by circRHOBTB3 overexpression or knockdown, However, we found a negative linear correlation between the expression level of circRHOBTB3 and miR-600 in 110 cases of PDAC tissues, suggesting that circRHOBTB3 function as miR-600 sponge without affecting the expression of miR-600 (Fig. 3l). More importantly, a Kaplan-Meier analysis revealed that lower miR-600 expression was associated with decreased OS in PDAC patients (Fig. 3m). Taking together, our results indicated that circRHOBTB3 functions as a miR-600 sponge in PDAC.

### MiR-600 abrogates the proliferation promoting effects by circRHOBTB3

**Fig. 4:**
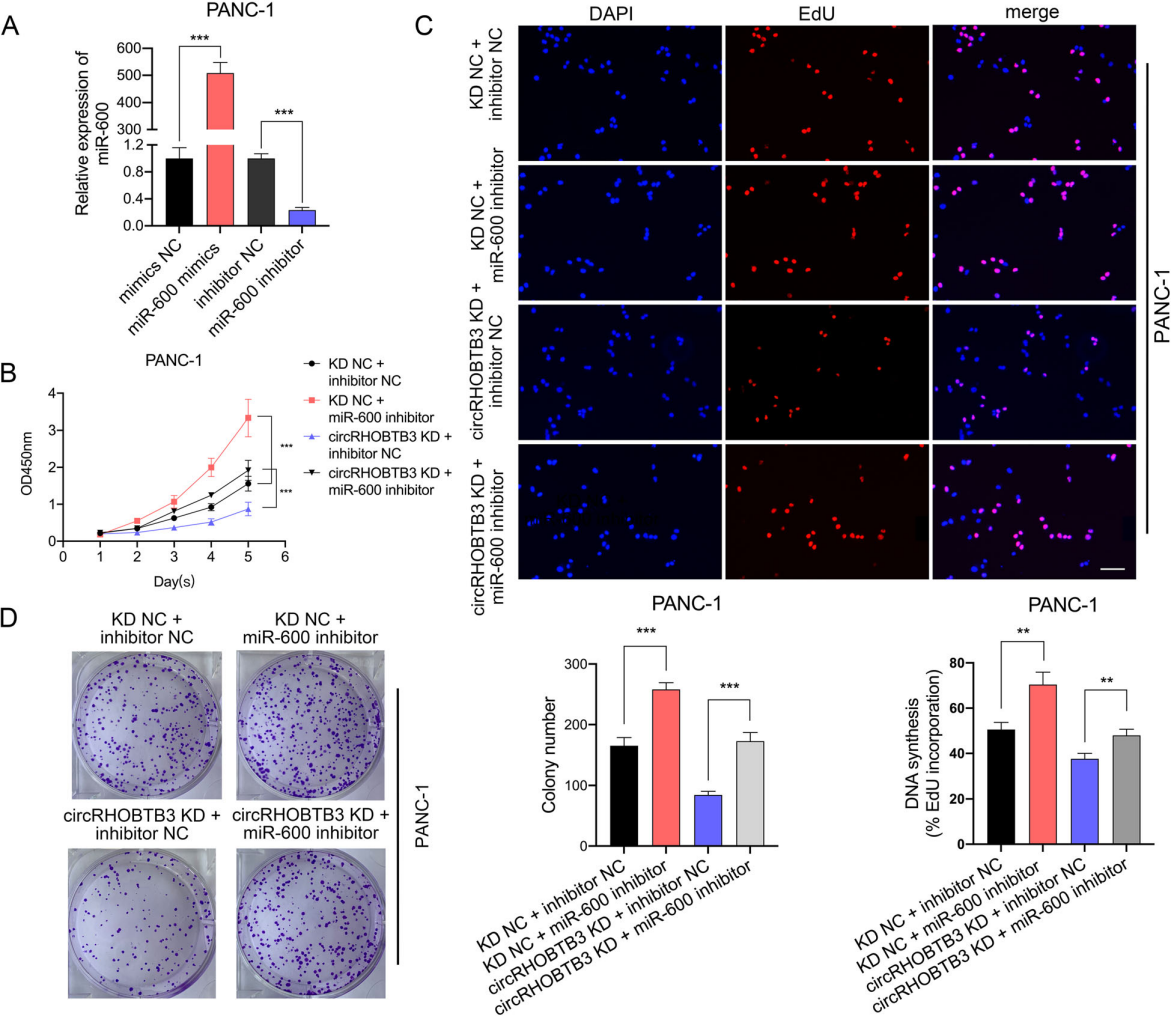
MiR-600 abrogates the proliferation promoting effect by circRHOBTB3 in PANC-1 cell line. **a** The efficiency of miR-600 mimics and inhibitor were examined by qRT-PCR in PANC-1 cell lines. **b-d** The CCK-8 assays, colony formation assays showed and Edu incorporation assays revealed that miR-600 inhibitor reversed the reduced effect of PANC-1 cell proliferation by circRHOBTB3 knockdown. The EdU samples were imaged at 200× magnification. Scale bar = 50 μm. All data are presented as the means ± SD of three independent experiments. *p < 0.05, **p < 0.01, ***p < 0.001

In order to further illustrating the underlying mechanism by which miR-600 regulating PDAC functions, we performed rescue experiments by co-transfecting PDAC cells with circRHOBTB3 KD lentivirus or overexpression plasmid along with miR-600 mimics and inhibitors. The efficiency of miR-600 mimics and inhibitor transfection on miR-600 expression level in PANC-1 was verified by qRT-qPCR (Fig. 4a). The results revealed that the miR-600 inhibitor significantly promoted the proliferation of PANC-1 and reversed the proliferation suppressive effects by circRHOBTB3 downregulation through CCK-8, EdU incorporation assays and colony formation (Fig. 4b-d). Similar results were observed in MiaPaca-2 cells transfected with miR-600 mimics and the circRHOBTB3 plasmid (Figure S2a-c). Collectively, these experiments demonstrated that miR-600 has an inhibitory effect on PDAC cells and may serve a crucial function downstream of circRHOBTB3.

### NACC1 is the direct downstream target of miR-600

**Fig. 5:**
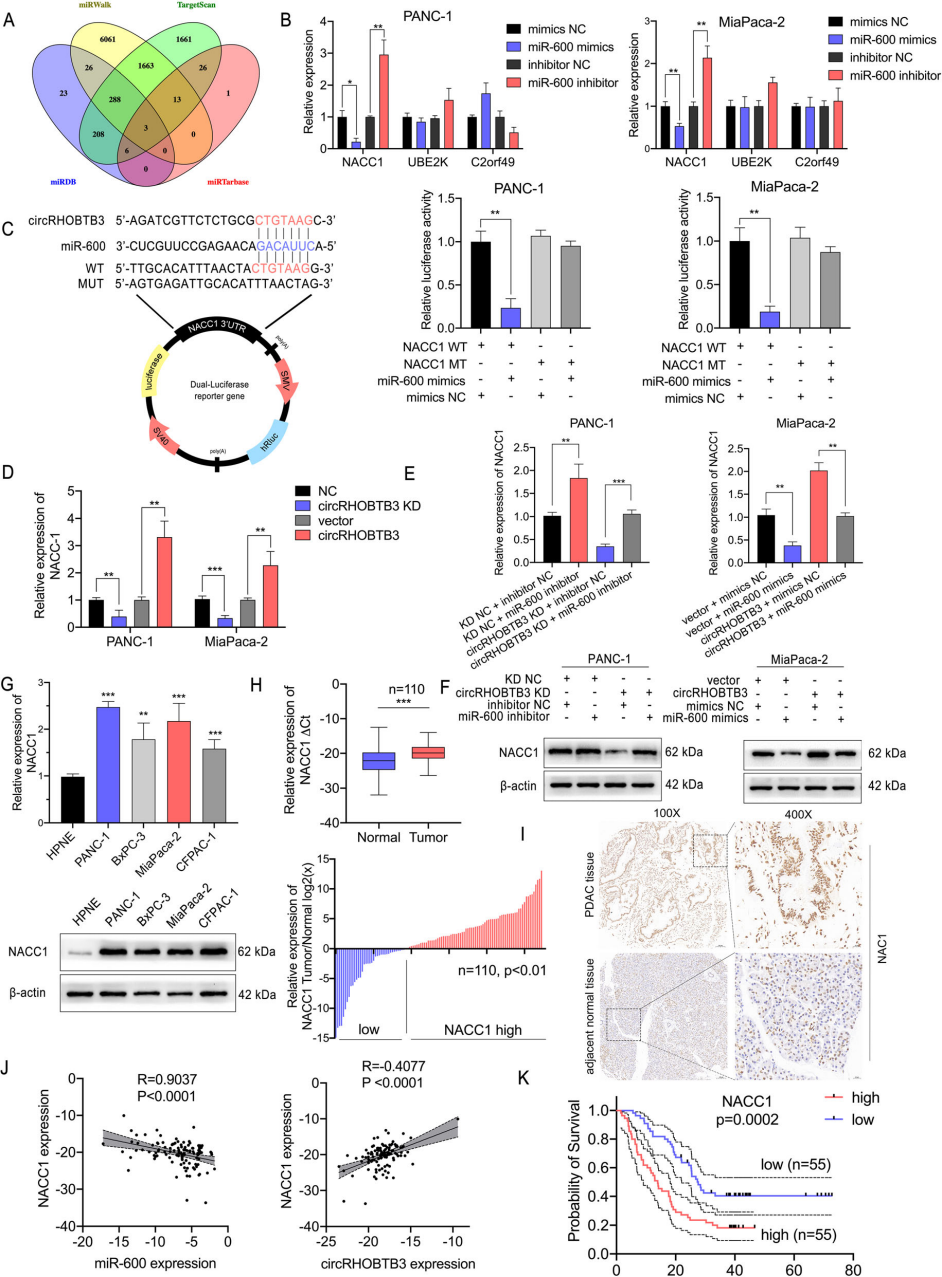
NACC1 is a direct target of miR-600, upregulated in PDAC cells and tissues and serves as a predictor for poor OS. **a** Venn diagram showing 3 potential target genes predicted by four databases (miRWalk, TargetScan, miRDB, and miRTarbase). **b** The three candidate target genes of miR-600 were validated by evaluating their expression upon transfection with miR-600 mimics or tis inhibitor. **c** The schematic diagram of NACC1 3’UTR wild-type (WT) and mutant (MT) luciferase reporter plasmid and the corresponding circRHOBTB3 sequence. The relative luciferase activities of the reporter were analyzed in PANC-1 and MiaPaca-2 cells co-transfected with miR-600 mimics or its control. **d** The expression of NACC1 mRNA in PANC-1 and MiaPaca-2 cells upon circRHOBTB3 overexpression or knockdown were examined by qRT-PCR. **e** The expression levels of NACC1 mRNA were measured by qRT-PCR with co-transfecting circRHOBTB3 knockdown lentivirus and miR-600 inhibitor or circRHOBTB3 overexpression vector and miR-600 mimics. **f** The protein levels of NAC1 were measured by western blotting with co- transfecting circRHOBTB3 knockdown lentivirus and miR-600 inhibitor or circRHOBTB3 overexpression vector and miR-600 mimics. **g** The expression of NACC1 in PDAC cell lines were determined by qRT-PCR and western blotting. **h** The expression of NACC1 in PDAC tissues and adjacent normal tissues were validated by qRT-PCR. **i** IHC staining of NACC1 in cancerous and adjacent normal tissues from PDAC patients. The samples were imaged at 100× and 400× magnification. Scale bar = 100 and 25 μm. **j** The Pearson correlation analysis revealed that expression level of NACC1 was negatively correlated with miR-600 but positively correlated with circRHOBTB3. **k** The Kaplan-Meier survival curve showed that patients with high NACC1 expression had poorer overall survival than the low expression group. All data are presented as the means ± SD of three independent experiments. *p < 0.05, **p < 0.01, ***p < 0.001

As illustrated in considerable literature, miRNAs interact with the 3’UTR region of target genes mRNA to induce its degradation by forming a RISC complex [23]. To further investigate the target genes of miR-600 in PDAC cells, we performed another bioinformatics analysis using online databases miRWalk (http://mirwalk.umm.uni-heidelberg.de/), TargetScan (http://www.targetscan.org/mamm_31/), miRDB (http://mirdb.org/) and miRTarbase (http://mirtarbase.cuhk.edu.cn/php/index.php) (Fig. 5a). Through overlapping the results obtained from these four subjects, we paid attention on three candidate genes most possibly targeted by miR-600 for further validation. qRT-PCR showed that only NACC1 expression was negatively regulated by miR-600 mimics or inhibitors consistently in pancreatic cancer cell lines (Fig. 5b). Next, to confirm that miR-600 directly bind to NACC1 3’UTR region, we constructed luciferase reporter plasmid comprising the 3’UTR of NACC1 mRNA. Luciferase reporter assays showed that the transfection with miR-600 mimics could significantly reduce the luciferase activity of the wild-type but not the mutant 3’UTR NACC1 construct in both cell lines (Fig. 5c). Notably, NACC1 shares the same binding region with circRHOBTB3 on miR-600, which indicating its role as a downstream target on circRHOBTB3-miR-600 axis. We performed qRT-PCR analysis and found that NACC1 expression is positively regulated by circRHOBTB3 (Fig. 5d). Furthermore, we detected NACC1 expression on both mRNA and protein levels upon circRHOBTB3 KD co-transfected with miR-600 inhibitor in PANC-1 cells. The results showed that NACC1 expression was downregulated by circRHOBTB3 knockdown but could be rescued by miR-600 inhibitor. The opposite regulation circumstance was validated in MiaPaca-2 cells with circRHOBTB3 overexpression vector and miR-600 mimics (Fig. 5e-f). Similar with circRHOBTB3, NACC1 is highly expressed in PDAC cell lines. qRT-PCR assay in human PDAC samples showed that NACC1 expression in cancerous tissue was relatively higher than that in normal tissue (Fig. 5g-h). Besides, the IHC staining of PDAC tissues and counterparts supported the same result on protein levels (Fig. 5i). Linear correlation analysis of the tissue expression level of NACC1, miR-600 and circRHOBTB3 suggested that NACC1 expression was negatively correlated with miR-600 but positively correlated with circRHOBTB3, which accorded with the circRHOBTB3/miR-600/NACC1 regulatory axis hypothesis (Fig. 5j). Moreover, patients with higher NACC1 expression levels had poorer OS, which indicated that NACC1 could also be a risk factor for overall survival in PDAC patients (Fig. 5k). Taking together, circRHOBTB3 restrained miR-600 and further induced NACC1 degradation, while both circRHOBTB3 and NACC1 may function as oncogene in PDAC.

### CircRHOBTB3 promotes PDAC proliferation via the miR-600/NACC1 axis

**Fig. 6:**
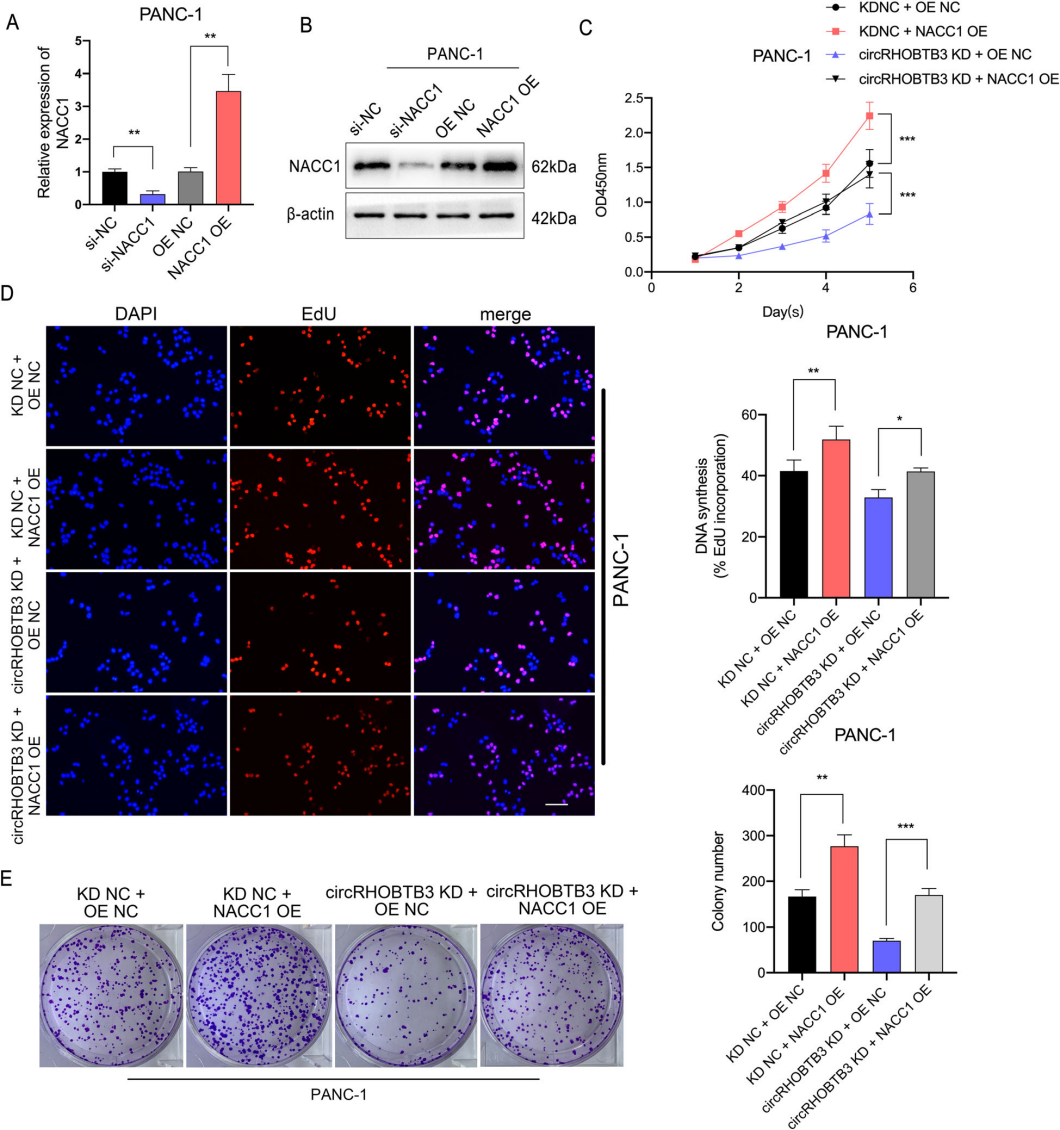
NACC1 overexpression restrained the suppression effect of PDAC by circRHOBTB3 knockdown. **a** The efficiency of NACC1 siRNA and overexpression vector is measured by qRT-PCR in PANC-1. **b** The protein levels of NACC1 knockdown and overexpression were determined by Western Blotting. **c-e** The retarded proliferation ability caused by circRHOBTB3 was reversed by NACC1 overexpression in PANC-1 cells in CCK-8, EdU incorporation and colony formation assays. All data are presented as the means ± SD of three independent experiments. *p < 0.05, **p < 0.01, ***p < 0.001

Given that NACC1 is the downstream target of miR-600 and circRHOBTB3, we further evaluated whether circRHOBTB3 promoted PDAC progression through NACC1. We transfected PANC-1 and MiaPaca-2 cell lines with NACC1 siRNA and overexpression plasmid. The efficiency of two constructs was validated by qRT-PCR and Western blot (Fig. 6a-b). Functional experiments showed that NACC1 overexpression in PANC-1 cells could promote cellular proliferation and colony formation, while the proliferation rate is largely reduced by NACC1 knockdown in MiaPaca-2 cells. Subsequently, we found that the inhibitory effect on cellular proliferation of circRHOBTB3 knockdown could be considerably reversed by NACC1 overexpression in PANC-1 cells, while the proliferation promoting effects of circRHOBTB3 overexpression could be retarded by NACC1 knockdown in MiaPaca-2 cells (Fig. 6c-e, Figure S3a-c). Taken together, our results indicated that circRHOBTB3 promotes the progression of PDAC via the miR-600/NACC1 axis.

### CircRHOBTB3 facilitates the autophagy response of PDAC cells via regulating NAC1 levels

**Fig. 7:**
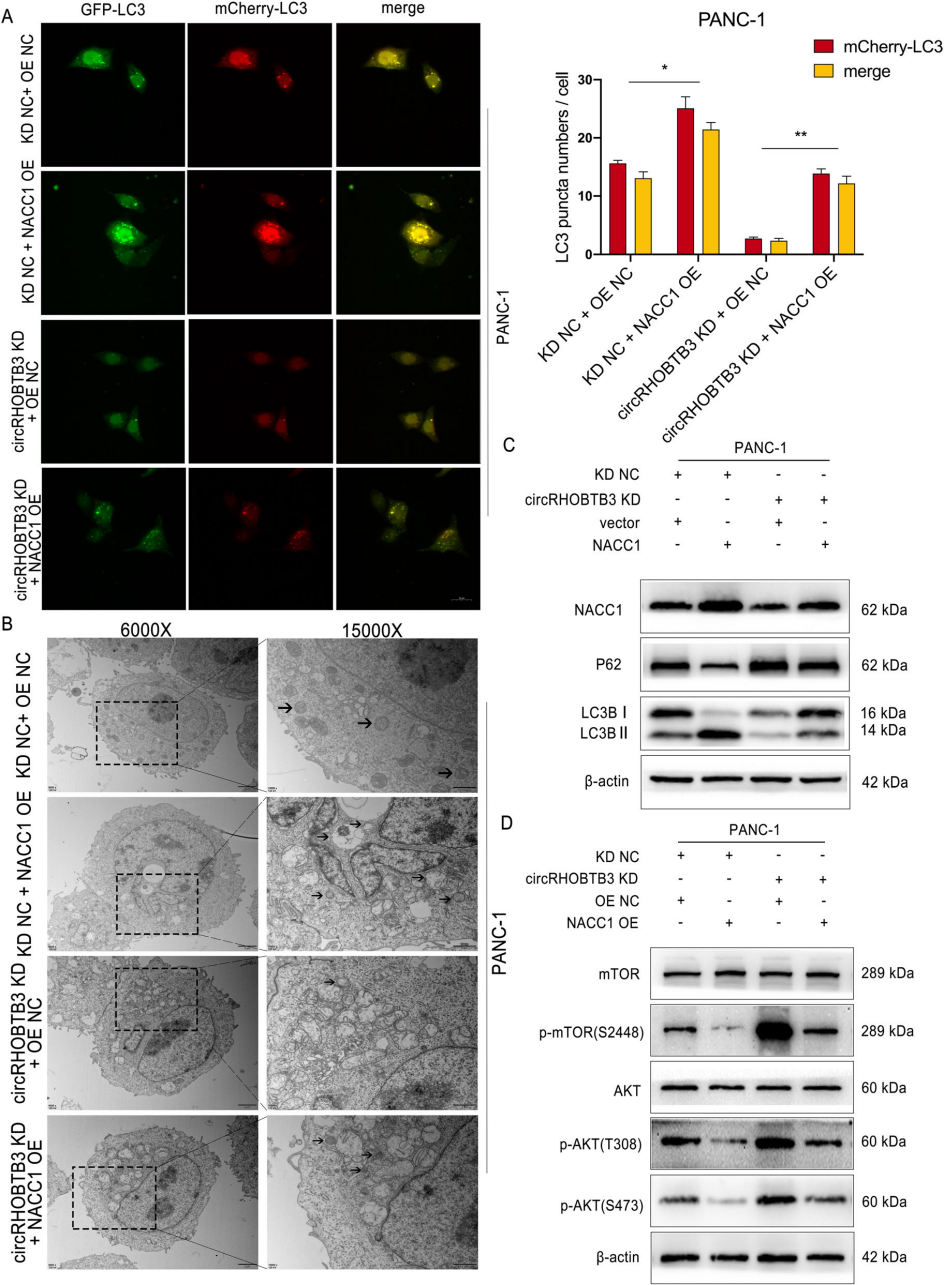
NACC1 overexpression reversed the inhibiting effect on PANC-1 cells autophagy by circRHOBTB3 knockdown. **a** mCherry-GFP-LC3B labeled PANC-1 cells were infected with circRHOBTB3 knockdown lentivirus and NACC1 overexpression vector, and the autophagy flux was analyzed by confocal microscopy. Representative confocal microscopy images and quantitative data were shown. Scale bar, 20 μm. **b** The autophagosome in PANC-1 cells were detected by transmission electron microscope (TEM) upon circRHOBTB3 knockdown and NACC1 overexpression and corresponding negative control groups. The samples were imaged at 6000X and 15,000X magnification. Arrows indicate autophagosomes in cells. **c-d** The protein levels of LC3B II, p62, phosphorylated AKT(p-AKT(T308), p-AKT(S473)), and phosphorylated mTOR(p-mTOR) in PANC-1 cells with circRHOBTB3 knockdown upon NACC1 overexpression. All data are presented as the means ± SD of three independent experiments. *p < 0.05, **p < 0.01

NAC1 has been reported to be associated with autophagy in ovarian cancer [16], we wondered whether it could mediate PDAC cells autophagy response for proliferation. Subsequently, the autophagy flux detection revealed that autophagy level was suppressed after circRHOBTB3 knockdown in PANC-1 cells. Moreover, the increased autophagy after NACC1 overexpression could be suppressed by circRHOBTB3 knockdown, which indicated that circRHOBTB3 could regulate autophagy level of PDAC cells via controlling NACC1 expression (Fig. 7a). On the other hand, we performed the opposite effect experiments in MiaPaca-2 cells and the results revealed the same that circRHOBTB3 could promote autophagy flux in PDAC cells via miR600/NACC1 axis (Figure S4a). Meanwhile, the transmission electron telescope showed that there were less autophagosomes upon circRHOBTB3 knockdown but could be reversed by NACC1 overexpression in PANC-1 cells, and NACC1 siRNA could restrain the autophagy promoting effect of circRHOBTB3 overexpression in MiaPaca-2 cells, which coordinated the results we had above (Fig. 7b, Figure S4b). Besides, PANC-1 cells with circRHOBTB3 knockdown showed higher p62 levels but lower LC3B II levels, while NACC1 overexpression could partially reverse the expression change of autophagy-related protein. On the contrary, the pro-autophagy role of circRHOBTB3 overexpression could be retarded by NACC1 knockdown with siRNA (Fig. 7c, Figure S4c). In the view of above, our experiments revealed that circRHOBTB3 could promote PDAC cells autophagy by upregulating NACC1.

### CircRHOBTB3 promotes proliferation of PDAC cells by increasing autophagy levels

**Fig. 8:**
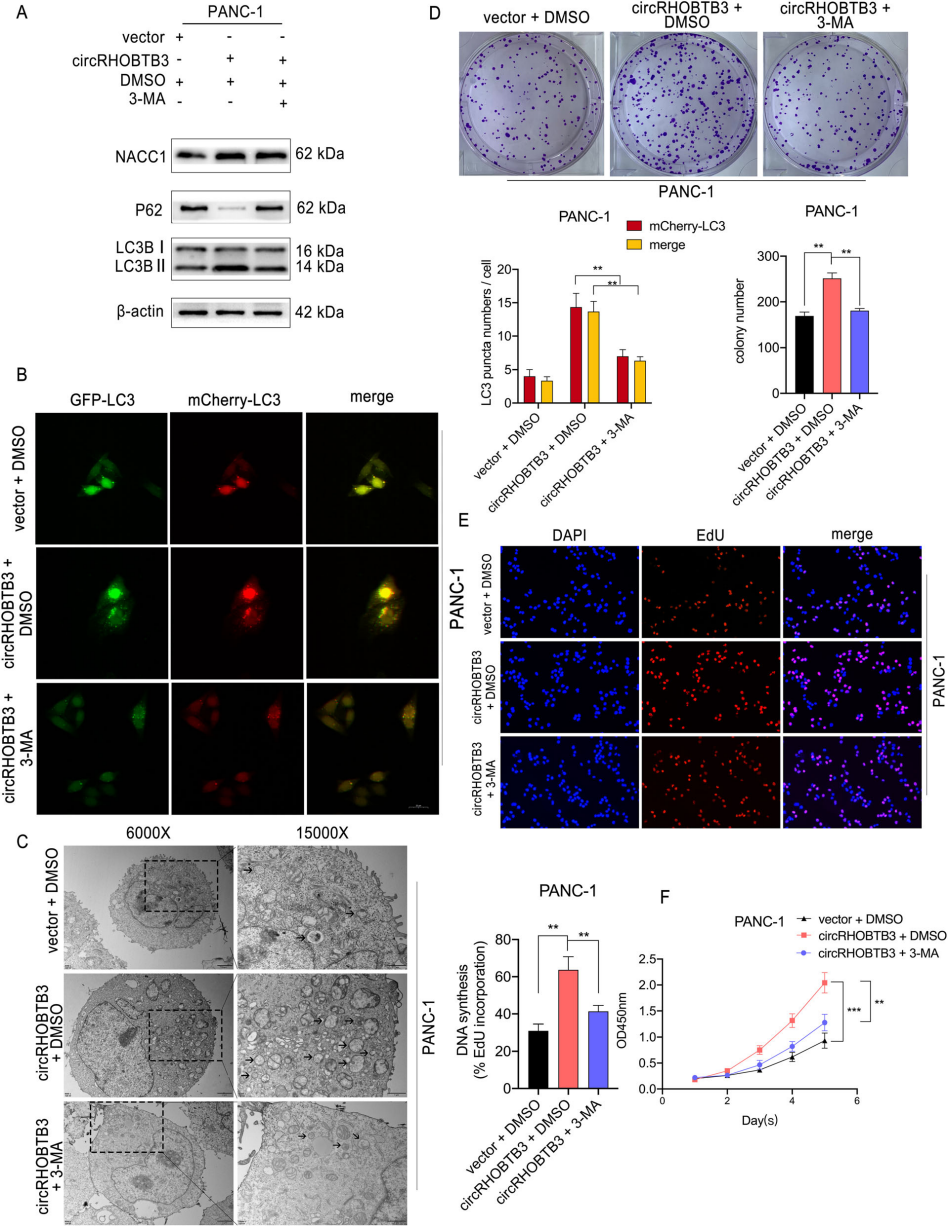
circRHOBTB3 promotes PANC-1 cell proliferation by activating autophagy response. **a** The protein level of p62 and LC3B were determined by western blotting in PANC-1 cells transfected circRHOBTB3 overexpressing vector with or without 3-Methyladenine (3-MA, 2.5 mM, 24 h) treatment. **b** The mCherry-GFP-LC3B labeled PANC-1 cells were transfected with circRHOBTB3 overexpression vector with or without 3-MA (2.5 mM, 24 h) treatment, the LC3B puncta were analyzed by confocal microscopy. Representative images and quantitative data were shown, with 20 μm scale bar. **c** The transmission electron microscopy revealed the autophagosome with the magnification of 6000X and 15,000X, Arrows indicate autophagosomes in cells. Scale bar 2 μm and 1 μm respectively. **d** The colony formation revealed that 3-MA (2.5 mM, 24 h) treatment inhibited the increased proliferation after circRHOBTB3 overexpression. **e** The DNA synthesis ability was restrained by 3-MA (2.5 mM, 24 h) treatment in cells with overexpressed circRHOBTB3 as suggested by EdU incorporation assays. **f** The growth curve of PANC-1 cells showed reversed proliferation capability upon 3-MA (2.5 mM, 24 h) treatment in cells with circRHOBTB3 overexpression. All data are presented as the means ± SD of three independent experiments. **p < 0.01, ***p < 0.001

Given that autophagy is an adaptive response upon inadequate energy supplying for PDAC progression. We wondered whether the proliferation promoting effects of circRHOBTB3 relies on regulation of autophagy. Consequently, we performed another rescue experiment using autophagy inhibitor 3-MA (3-methyladenine) with circRHOBTB3 overexpression. We found that 3-MA treatment reversed the effects of circRHOBTB3 on autophagy-associated protein levels in PANC-1 and MiaPaca-2 cells (Fig. 8a, Figure S5a. Next, we transfected mCherry-GFP-LC3B labeled PDAC cells with circRHOBTB3 plasmid and 3-MA, and observed that the red and yellow LC3 puncta were increased in PDAC cells after circRHOBTB3 overexpression but restrained by 3-MA applying (Fig. 8b, Figure S5b). Besides, the transmission electron microscope showed that 3-MA successfully inhibited the augmented autophagy levels in PDAC cells with circRHOBTB3 overexpression (Fig. 8c, Figure S5c). On the other hand, we found that 3-MA treatment reversed the promotive effects of circRHOBTB3 overexpression on PANC-1 and MiaPaca-2 cell proliferation (Fig. 8d-f, Figure S5d-f). Collectively, these data indicated that circRHOBTB3 promotes PDAC cell proliferation by accelerating autophagy response.

### CircRHOBTB3 regulates PDAC autophagy level via Akt/mTOR pathways

**Fig. 9:**
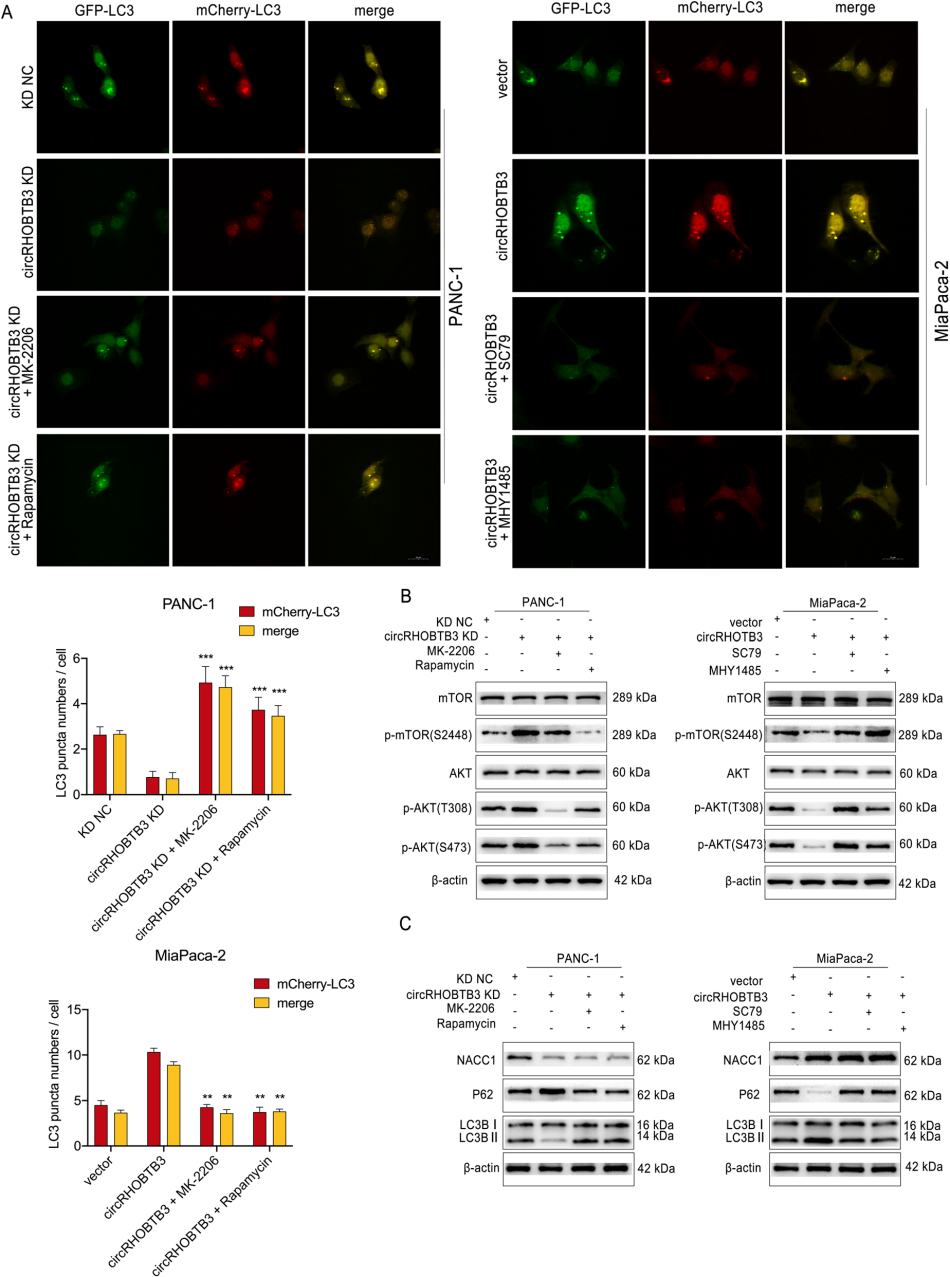
circRHOBTB3 promotes PDAC cell autophagy response via Akt/mTOR pathway. **a** mCherry-GFP-LC3B labeled PANC-1 were infected with circRHOBTB3 knockdown lentivirus and overexpression vector respectively, and treated with MK-2206 (5 μM,48 h) or Rapamycin (50 nM, 48 h), while circRHOBTB3 overexpression vector and SC79 (2.5 μg/ml, 48 h) or MHY1485 (5 μM, 48 h) were used for MiaPaca-2 cells. The dose of inhibitors and activators was twice as PANC-1 cells on which treated with MiaPaca-2 cells. Autophagy flux was analyzed by confocal microscopy. Representative confocal microscopy images and quantitative data were shown, with the 20 μm scale bar. **b** Activation or deactivation of signaling pathways were validated by detecting the protein phosphorylation level after Akt/mTOR activators or inhibitors treatment with western blot. **c** The protein expression levels of NACC1, LC3B I/II and p62 were detected by western blotting. All data are presented as the means ± SD of three independent experiments. **p < 0.01, ***p < 0.001

As reported in various literature, Akt/mTOR pathway is a common pathway regulating PDAC tumor progression, and acts as an inhibitory role in autophagy response [24, 25]. Consequently, we conducted western blot and the results revealed that circRHOBTB3 knockdown increased protein phosphorylation levels but not the expression of Akt and mTOR. These effects could be reversed by NACC1 overexpression in PANC-1 cells. On the opposite, MiaPaca-2 cells showed decreased activation of mTOR and Akt signaling upon circRHOBTB3 overexpression, which could be rescued by NACC1 knockdown (Fig. 7d, Figure S4d). To further examine whether circRHOBTB3 promotes autophagy in PDAC cells by inhibiting Akt/mTOR signaling axis, circRHOBTB3 knockdown cells were treated with Akt phosphorylation inhibitor MK-2206 or mTOR phosphorylation inhibitor Rapamycin. We observed that MK-2206 and Rapamycin treatment alleviated the inhibitory effects of circRHOBTB3 on autophagy flux in PANC-1 and MiaPaca-2 cell lines (Fig. 9a). In parallel to decreased phosphorylation levels of Akt and mTOR, p62 protein decreased while LC3B II increased by MK-2206 or Rapamycin treatment in both cell lines (Fig. 9b, c). On the other hand, the Akt signaling activator SC79 and mTOR signaling activator MHY1485 displayed the opposite effects in MiaPaca-2 cells with circRHOBTB3 overexpression (Fig. 9a). These data confirmed that circRHOBTB3 facilitates PDAC cells autophagy by inhibiting Akt/mTOR signaling axis.

### FUS-mediated circRHOBTB3/miR600/NACC1 axis is corelated with PDAC prognosis

**Fig. 10:**
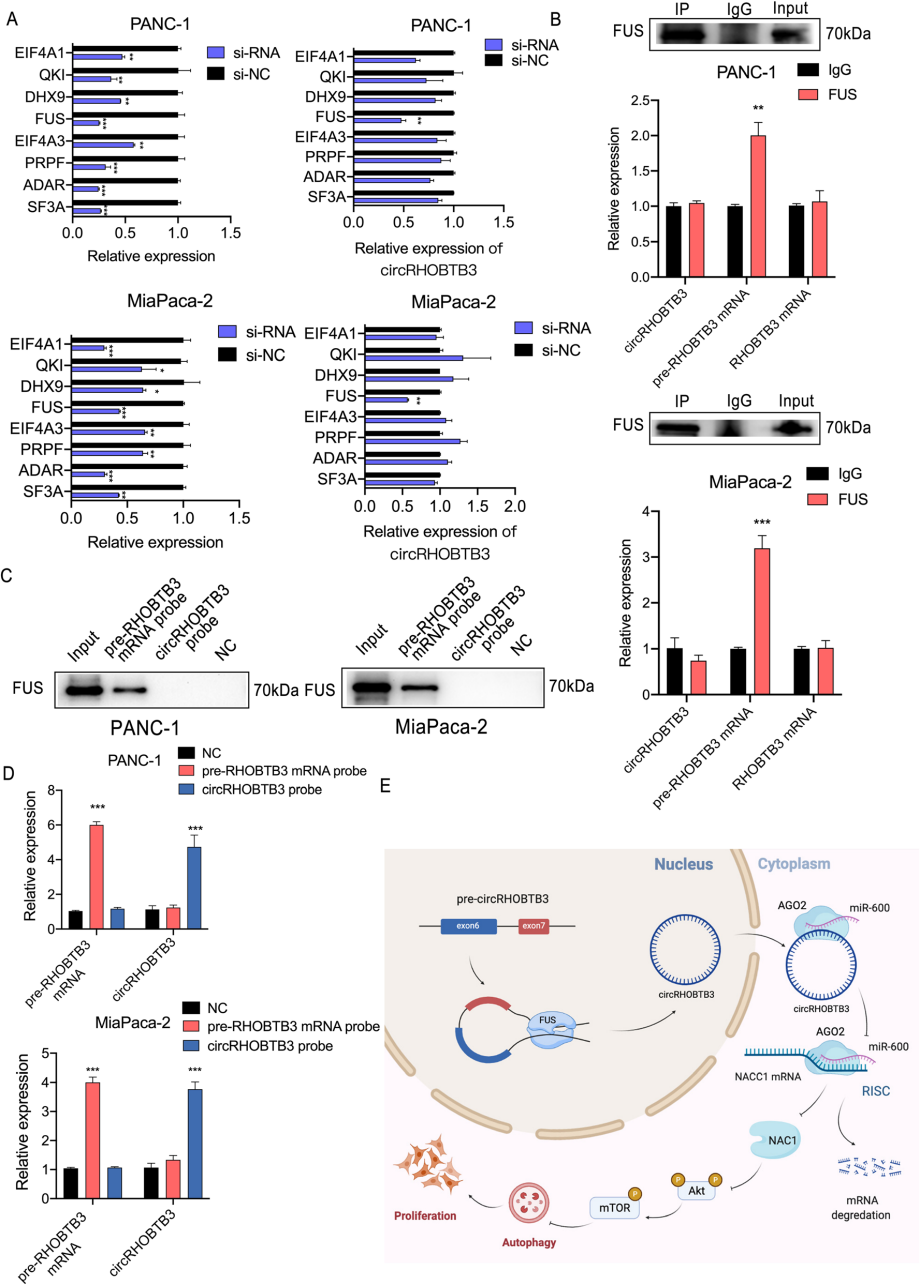
FUS binds to pre-RHOBTB3 mRNA and mediates the biogenesis of circRHOBTB3. **a** The efficiency of 8 candidate biogenesis-regulating RNA-binding proteins siRNA and relative expression of circRHOBTB3 upon 8 siRNAs transfection compared with negative control. **b** The anti-FUS RIP assays exhibited that FUS antibody successfully precipitated FUS protein as well as pre-RHOBTB3 mRNA rather than circRHOBTB3, neither RHOBTB3 mRNA in PANC-1 and MiaPaca-2 cell lines. **c** The pull-down assays were conducted applying pre-RHOBTB3 mRNA probe, circRHOBTB3 probe and negative control probe for further confirmation of the binding relationship between FUS and pre-RHOBTB3. The results revealed that only the pre-RHOBTB3 mRNA probe could successfully pull down FUS protein, verified by western blotting. **d** The efficiency and specificity of each probe were validated by qRT-PCR **e** Schematic illustration indicates the mechanism by which circRHOBTB3 promotes autophagy to facilitate malignant progression of PDAC cells via miR-600/NACC1/Akt/mTOR pathway. All data are presented as the means ± SD of three independent experiments. **p < 0.01, ***p < 0.001

Once illustrated that circRHOBTB3 regulates PDAC progression via miR600/NACC1 axis, we wondered what the initial factors and the upstream regulatory mechanism of circRHOBTB3 in PDAC are. Firstly, we identified 8 candidate circRNA biogenesis-associated RNA-binding proteins (EIF4A, QKI, DHX9, FUS, EIF4A3, PRPF, ADAR and SF3A), and constructed siRNAs for all of them. The efficiency of each siRNA was confirmed by qRT-PCR. The results showed that circRHOBTB3 expression was significantly reduced only when the RNA-binding protein FUS was downregulated, circRHOBTB3 expression exhibited reduced levels, indicating that FUS could be a mediating protein that promotes circRHOBTB3 biogenesis (Fig. 10a). As FUS is a DNA/RNA-binding protein that plays a role in various cellular processes such as transcription regulation, RNA splicing, RNA transport, DNA repair and damage response [26], it also binds to nascent pre-mRNAs and acts as a molecular mediator between RNA polymerase II and U1 small nuclear ribonucleoprotein thereby coupling transcription and splicing [27]. Therefore, it has been reported that FUS controlled back-splicing reactions leading to circRNA production [9]. Then we assumed that whether FUS could bind to pre-RHOBTB3 mRNA and then promote the back-splicing process of circRHOBTB3 production. We performed RNA immunoprecipitation (RIP) assays and found that FUS antibody could bind to pre-RHOBTB3 mRNA but not circRHOBTB3 or RHOBTB3 mRNA (Fig. 10b). On the other hand, pull-down assays results conformed to above that the pre-RHOBTB3 mRNA probe could precipitate FUS protein in PANC-1 and MiaPaca-2 cells (Fig. 10c). Taken together, we primarily validated the biogenesis promoting role of FUS in circRHOBTB3 biogenesis in PDAC cells. Given the discovery of the circRHOBTB3/miR-600/NACC1 axis in PDAC cells and that the characteristics of each subject have been illustrated, we next evaluated the clinical significance of circRHOBTB3, miR-600 and NACC1 in a cohort of 110 patients. Patients were divided into high and low groups based on the median expression. The relationship between circRHOBTB3, miR-600 and NACC1 expression and the clinical characteristics of PDAC patients are listed in Table 1. The results revealed that tumor size, vascular invasion, and clinical stage, especially T stage were significantly associated with at least one of these three genes. Further univariate and multivariate Cox regression analysis showed that circRHOBTB3, miR-600 and NACC1 expression levels were independent prognostic factors for PDAC patients, as were the tumor size and clinical stages (Table 2). Thereupon, we had a conclusion that FUS-mediated circRHOBTB3/miR-600/NACC1 axis is correlated with PDAC prognosis.

**Table 1:** Association of circRHOBTB3, miR-600, NACC1 expression with the clinicopathological features of 110 PDAC patients

Characteristics	circRHOBTB3 expression level			miR-600 expression level			NACC1 expression level		
	High	Low	***P***-value	High	Low	***P***-value	High	Low	***P***-value
**Total cases**	55	55		55	55		55	55	
**Gender**									
Male	38	37	0.838	39	36	0.539	38	37	0.838
Female	17	18		16	19		17	18	
**Age (year)**									
< 60	41	32	0.069	32	41	0.069	40	33	0.158
≥ 60	14	23		23	14		15	22	
**Serum CA19–9(U/mL)**									
CA19–9 < 39	5	14	0.076	11	8	0.545	6	13	0.209
39 ≤ CA19–9 < 1000	38	31		35	34		36	33	
CA19–9 ≥ 1000	12	10		9	13		13	9	
**Location**									
Head	36	37	0.84	36	28	0.738	37	37	1
Body/tail	19	18		19	17		18	18	
**Diameter (cm)**									
≤ 4	36	48	0.007**	47	37	0.025*	32	51	< 0.0001***
> 4	19	7		8	18		23	4	
**Differentiation**									
Poor/moderate	36	33	0.554	33	36	0.554	33	35	0.695
Well	19	22		22	19		22	20	
**Vascular invasion**									
Present	44	33	0.022*	30	43	0.009**	40	35	0.306
Absent	11	22		25	12		15	20	
**Nerve invasion**									
Present	52	47	0.112	49	49	1	49	50	0.751
Absent	3	8		6	6		6	5	
**T stage**									
T1/T2	24	36	0.022*	36	24	0.022*	26	33	0.181
T3/T4	31	19		19	31		29	22	
**N stage**									
N0	21	26	0.335	26	21	0.335	26	21	0.335
N1/N2	34	29		29	34		29	34	
**M stage**									
M0	54	54	1	54	54	1	55	53	0.154
M1	1	1		1	1		0	2	
**Clinical stage**									
I-IIa	18	20	0.688	25	13	0.016*	19	19	1
IIb-IV	37	35		30	42		36	36	
**Postoperative Recurrence or liver metastasis**									
Present	31	31	1	33	31	0.699	33	30	0.563
Absent	24	24		22	24		22	25	

All data are presented as the mean ± SD. *p < 0.05, **p < 0.01, ***p < 0.001

**Table 2:** Univariate and multivariate analysis of prognostic factors in PDAC patients (*n* = 110)

Variables	Univariate analysis				Multivariate analysis		
	Cases	Events	Median survival (months)	***P*** value	HR	95%CI	***P*** value
Gender, male/ female	75/ 35	53/ 24	20.0/ 19.2	0.850			
Age, < 60/ ≥60 (year)	37/ 73	21/ 56	27.4/ 18.2	0.027*			0.069
Serum CA199 (U/ml)							
< 39, ≥39	19/ 69	11/ 50	28.4/ 19.3	0.097			
≥ 39, ≥1000	69/ 22	50/ 16	19.3/ 14.1	0.550			
< 39, ≥1000	19/ 22	11/ 16	28.4/ 14.1	0.061			
Location, head/ body or tail	73/ 37	51/ 26	20.0/ 18.5	0.797			
Diameter, ≤4/ > 4 (cm)	84/ 26	53/ 24	25.2/ 9.1	< 0.001***			0.407
Differentiation, poor,moderate/ well	69/ 41	48/ 29	20.0/ 19.2	0.968			
Microscopic vascular invasion,absent/ present	33/ 77	19/ 58	30.3/ 18.2	0.027*			0.109
Microscopic nerve invasion, absent/ present	11/ 99	8/ 69	19.3/ 20.0	0.848			
T stage, T1, 2/ T3, 4	60/ 50	36/ 41	26.8/ 12.9	0.001**	1.728	1.447–3.869	0.025*
N stage, N0/ N1,2	47/ 63	30/ 47	25.3/ 18.2	0.109			
M stage, M0/ M1	108/ 2	75/ 2	19.3/ 22.0	0.816			
Clinical stage, I-IIa/ IIb-IV	38/ 72	19/ 58	34.1/ 17.7	< 0.001***	2.661	1.513–4.678	0.001**
circRHOBTB3 expression, low/ high	55/ 55	34/ 43	25.4/ 14.2	0.005**	2.700	1.636–4.456	< 0.001***
miR-600 expression, low/ high	55/ 55	51/ 26	26.1/ Undefined	< 0.001***	0.179	0.103–0.309	< 0.001***
NACC1 expression, low/ high	55/ 55	32/ 45	27.6/ 13.9	< 0.001***	2.366	1.447–3.869	0.001**

## Discussion

During the past decades, circRNAs research has been through fundamental variation. CircRNAs were once considered noise generated by transcription, with no significant biological function [28]. However, with the evolution of high-throughput sequencing, there are variety functional circRNAs coming forward [4]. CircRNAs have unique functions in regulation of gene expression and play important roles in many different types of cancers [29, 30]. For example, several circRNAs function as miRNA sponge to regulate downstream target genes expression by form a RISC complex mediating mRNA degradation [31]. Besides, they can also bind to specific proteins to influence their functions of encode peptides [32, 33]. Nevertheless, how circRNAs contribute to PDAC biological process remain largely unknown, and warrant further exploration.

In the present study, we conducted high-throughput sequencing to profile circRNA expression in 3 pairs of PDAC tumor tissues and adjacent normal tissues. Further confirmation and experiments using PDAC cells and tissues illustrated that circRHOBTB3 is significantly upregulated in PDAC tissues as well as cell lines. Moreover, circRHOBTB3 is associated with the poor prognosis of PDAC patients. Gain and loss of function experiments showed that circRHOBTB3 promotes PDAC cells proliferation in vivo and in vitro, indicating its oncogenic role in PDAC and its potential as a biomarker for prediction of PDAC patients.

Accumulating evidence have revealed that circRNAs regulate cellular function as miRNA sponges. Thomas. et al. found that ciRS-7 functioned as a sponge of miR-7, resulting in increased levels of miR-7 targets [21]. Chen. et al. reported that circNFIB1 acted as a miRNA sponge and inhibited lymphangiogenesis in pancreatic cancer [34]. Herein, with bioinformatics analysis in three different databases, we performed RNA pull-down assays showed that circRHOBTB3 interacted with miR-600. Luciferase reporter assays validated the sponge effect of circRHOBTB3 on miR-600 and further confirmed the binding sites on circRHOBTB3. However, it seemed contradictory that in 110 PDAC patients, circRHOBTB3 expression level is negatively associated with miR-600 expression, while knocking down or overexpressing circRHOBTB3 barely affected miR-600 levels in PDAC cells. Based on this phenomenon, we hypothesized that the stable expression pattern of circRHOBTB3 and miR-600 in tissues might be dependent on not each other but the malignancy of tumor itself. We also wondered that why circRHOBTB3 could preferentially sponge miR-600 but no other proteins or RNAs and whether AGO2-binding increased the affinity of circRHOBTB3 to miR-600. For further research, we will continue investigating the potential protein binding pattern of circRHOBTB3 and the expression of circRHOBTB3 upon miR-600 variation in PDAC cells. In addition, rescue experiments showed that the circRHOBTB3 knockdown-induced suppression of colony formation, proliferation, and EdU incorporation could be rescued using an miR-600 inhibitor. Our results provided evidence to support the view that circRHOBTB3 binds to miR-600, acting as “miRNA sponge”, which is essential to the progression of PDAC.

NAC1, encoded by the NACC1 gene, promotes autophagy response, disables cellular senescence and binds to actin to regulate cancer cell cytokinesis [16, 35]. We demonstrated that NACC1 was a new target gene of miR-600. Moreover, functional studies demonstrated that circRHOBTB3 accelerated autophagy and promoted PDAC cell proliferation. Besides, NACC1 siRNA could restrained the positive effect of circRHOBTB3 on autophagy, indicating that circRHOBTB3 promotes PDAC autophagy levels through regulating NACC1 expression.

Next, considering the bidirectional function role of autophagy in cancer progression, we wondered whether increased autophagy level contributes to PDAC progression regulated by circRHOBTB3. For further detection of autophagy levels, we chose PI3K-III inhibitor 3-Methyladenine(3-MA), which could suppress the sequestration of autophagosome in an upstream manner. Then, we performed rescue experiments applying 3-MA and the results revealed that 3-MA could retard the proliferation accelerating effects of circRHOBTB3 overexpression. Taken together, our study firstly linked circRHOBTB3 with NACC1, autophagy, and tumor progression.

As published as a classic regulatory pathway, the Akt/mTOR phosphorylation pathway could significantly inhibit ULK1 and Beclin1 and then restrain autophagosome sequestration, which plays an upstream regulatory role in autophagy response [24, 25]. Consistently, our study revealed that circRHOBTB3 knockdown activated Akt and mTOR phosphorylation and thus reduced PDAC cell autophagy level. The opposite function experiments revealed the consistent results. Furthermore, blocking or activating Akt and mTOR phosphorylation using small-molecule compounds reversed the effect of circRHOBTB3 on autophagy, indicating that circRHOBTB3 promotes autophagy by via the NACC1/Akt/mTOR pathway in PDAC cells.

Besides, how circRHOBTB3 derived from its parental gene attracted our attention. The main hypothesis of back-splicing is that loop of the intron sequences flanking the downstream splice-doner site, and the upstream splice-acceptor site brings these sites into proximity [36]. This mechanism can be mediated by base pairing between inverted repeat elements namely Alu elements [7], or by dimerization of RNA-binding proteins like HQK encoded by QKI [8] or FUS [9] that binds to specific motifs in the flanking introns. Also, double-stranded RNA (dsRNA)-specific adenosine deaminase (ADAR) enzymes, and ATP-dependent RNA helicase A (DHX9) suppressed the biogenesis of circRNAs [37, 38]. In our study, we identified FUS, which could bind to pre-RHOBTB3 mRNA and thus promoted the biogenesis of circRHOBTB3, primarily illustrating the upstream manner of circRHOBTB3 in PDAC. However, through database prediction, we found that FUS could not only bind to pre-RHOBTB3 mRNA flanking regions but also circRHOBTB3 itself in Circular RNA Interactome. In the coming future, we aim to define the specific binding site on pre-RHOBTB3 mRNA as well as the domain on FUS and demonstrate the definite mechanism of the regulatory effect of FUS on circRHOBTB3 biogenesis.

Importantly, the Kaplan-Meier analysis revealed that high circRHOBTB3 expression, low miR-600 expression and high NACC1 expression were associated with the poor overall survival (OS) of 110 PDAC patients. And the circRHOBTB3/miR-600/NACC1 axis was associated with tumor size, vascular invasion and T stage of patients, and each element was independent prognostic factors for PDAC patients, foreboding that the circRHOBTB3/miR-600/NACC1 is correlated with PDAC prognosis. CircRHOBTB3 could serve as a potential therapeutic target for PDAC patients.

Finally, our study revealed that circRHOBTB3/miR-600/NACC1 axis promotes PDAC progression by accelerating autophagy response of PDAC cells via inhibiting Akt/mTOR pathway. Also, our research exhibits that circRHOBTB3 regulates PDAC behavior and may have important clinical implications and applications.

## Conclusion

In summary, we identified a novel circRNA induced by FUS, circRHOBTB3, that aberrantly inhibits NACC1/Akt/mTOR signaling by acting as a molecular sponge for miR-600, which subsequently promotes autophagy for PDAC proliferation. Our research provides a novel insight into the mechanism underlying circRNA-induced, autophagy-associated progression of PDAC and could lead to the development of a potential biomarker and therapeutic target for PDAC multi-therapy.

## Supplementary Information

**Additional file 1: Table S1.** Primers, probes and siRNAs used in the present research.

**Additional file 2: Table S2.** Antibodies and inhibitors/activators used in the present research.

**Additional file 3: Table S3.** Fifty-seven candidate miRNAs targeted by circRHOBTB3 predicted by overlapping three miRNA target databases results.

**Additional file 4: Figure S1.** CircRHOBTB3 promotes PDAC cells proliferation in vitro. **a.** Colony formation assays showed that circRHOBTB3 facilitated PDAC cell proliferation. **b.** EdU assays in PANC-1 and MiaPaca-2 cells were performed to evaluate cell proliferation capabilities. The samples were imaged at 200× magnification. Scale bar = 50 μm. All data are presented as the means ± SD of three independent experiments. *p < 0.05, **p < 0.01, ***p < 0.001.

**Additional file 5: Figure S2.** MiR-600 reverses the proliferation promotive effects by circRHOBTB3 overexpression in MiaPaca-2 cell line. a. The efficiency of miR-600 mimics and inhibitor were examined by qRT-PCR in MiaPaca-2 cell lines. **b-d.** Colony formation, EdU corporation assays and CCK-8 revealed that miR-600 abrogated the promoting role of circRHOBTB3 in MiaPaca-2 cell proliferation, The EdU samples were imaged at 200× magnification. Scale bar = 50 μm. All data are presented as the means ± SD of three independent experiments. *p < 0.05, **p < 0.01, ***p < 0.001.

**Additional file 6: Figure S3.** NACC1 knockdown reverses the oncogenic effect induced by circRHOBTB3 overexpression. **a.** The efficiency of NACC1 siRNA and overexpression vector is measured by qRT-PCR in MiaPaca-2. **b.** The protein levels of NACC1 knockdown and overexpression were determined by Western Blotting in MiaPaca-2 cell lines. **c-e.** MiaPaca-2 cells were divided into four groups (circRHOBTB3 vector + NACC1 si-NC, vector + NACC1 siRNA, circRHOBTB3 + NACC1 si-NC and circRHOBTB3 + NACC1 siRNA). The proliferation capabilities of MiaPaca-2 cells were detected through CCK-8, EdU corporation assays, colony formation assays. The EdU samples were imaged at 200× magnification. Scale bar = 50 μm. All data are presented as the means ± SD of three independent experiments. *p < 0.05, **p < 0.01, ***p < 0.001.

**Additional file 7: Figure S4.** circRHOBTB3 promotes MiaPaca-2 cell autophagy by indirectly regulating NACC1 expression. **a.** The mCherr-GFP-LC3B labeled MiaPaca-2 cells were transfected circRHOBTB3 overexpression plasmid and NACC1 siRNA, and the autophagy flux was analyzed by confocal microscopy. Representative confocal microscopy images and quantitative data were shown. Scale bar, 20 μm. **b.** The autophagosome in MiaPaca-2 cells were detected by transmission electron microscope (TEM) upon circRHOBTB3 overexpression and NACC1 knockdown by siRNAs and corresponding negative control groups. The samples were imaged at 6000× and 15,000× magnification. The arrows indicate autophagosomes in PDAC cells. **c-d.** The protein levels of LC3B II, p62, phosphorylated AKT(p-AKT(T308), p-AKT(S473)), and phosphorylated mTOR(p-mTOR) in MiaPaca-2 cells with circRHOBTB3 overexpression upon NACC1 knockdown. All data are presented as the means ± SD of three independent experiments. **p < 0.01.

**Additional file 8: Figure S5.** circRHOBTB3 promotes MiaPaca-2 cell proliferation by activating autophagy response. **a.** The protein level of p62 and LC3B were determined by western blotting in MiaPaca-2 cells transfected circRHOBTB3 overexpressing vector and 3-MA (5 mM, 24 h). **b.** The mCherry-GFP-LC3B labeled MiaPaca-2 cells were transfected with circRHOBTB3 overexpression vector and 3-MA (5 mM, 24 h), the LC3B puncta were analyzed by confocal microscopy. Representative images and quantitative data were shown, with 20 μm scale bar. **c.** The transmission electron microscopy of MiaPaca-2 cells revealed the autophagosome with the magnification of 6000× and 15,000×, scale bar 2 μm and 1 μm respectively. The arrows indicate autophagosomes in cells. **d.** The colony formation revealed that 3-Methyladenine (3-MA, 5 mM, 24 h) inhibited the increased proliferation ability by circRHOBTB3 overexpression. **e.** The DNA synthesis ability was restrained by 3-MA (5 mM, 24 h) on circRHOBTB3 overexpression via EdU incorporation assays. **f.** The growth curve of MiaPaca-2 cells showed reversed proliferation capability upon 3-MA (5 mM, 24 h) treatment. All data are presented as the means ± SD of three independent experiments. *p < 0.05, **p < 0.01, ***p < 0.001.

## Data Availability

All data generated or analyzed during this study are included in this published article.

## Abbreviations

circRNA: Circular RNA
PDAC: Pancreatic ductal adenocarcinoma
qRT-PCR: Quantitative reverse transcription polymerase chain reaction
RIP: RNA immunoprecipitation
FISH: Fluorescent in situ hybridization
NACC1: Nucleus accumbens associated 1
miRNA: Micro-RNA
RBPs: RNA binding proteins
3’UTR: 3′-untranslated regions
RISC: RNA-induced silencing complex
RHOBTB3: Rho-related BTB domain containing 3
DAPI: 4,6-diamidino-2-phenylindole
CCK-8: Cell count kit-8
EdU: 5-ethynyl-20-deoxyuridine
AGO2: Argonaute-2
siRNA: Short interfering RNA
IHC: Immunohistochemistry
DMEM: Dulbecco’s modified Eagle’s medium
FBS: Fetal calf serum
3-MA: 3-Methyladenine
OS: Overall survival
ADAR: Adenosine deaminases acting on RNA 1
EIF4A1: Eukaryotic initiation factor 4A-I
QKI: Protein quaking
DHX9: ATP-dependent RNA helicase A
SF3A1: Splicing factor 3A submit 1
PRPF8: Pre-mRNA-process-splicing factor 8
EIF4A3: Eukaryotic initiation factor 4A-III
