# Supplementary figures and images for: FUS-induced circRHOBTB3 facilitates cell proliferation via miR-600/NACC1 mediated autophagy response in pancreatic ductal adenocarcinoma
Source: J Exp Clin Cancer Res. 2021 Aug 20;40:261. doi: 10.1186/s13046-021-02063-w (PMC8377879; doi:10.1186/s13046-021-02063-w)

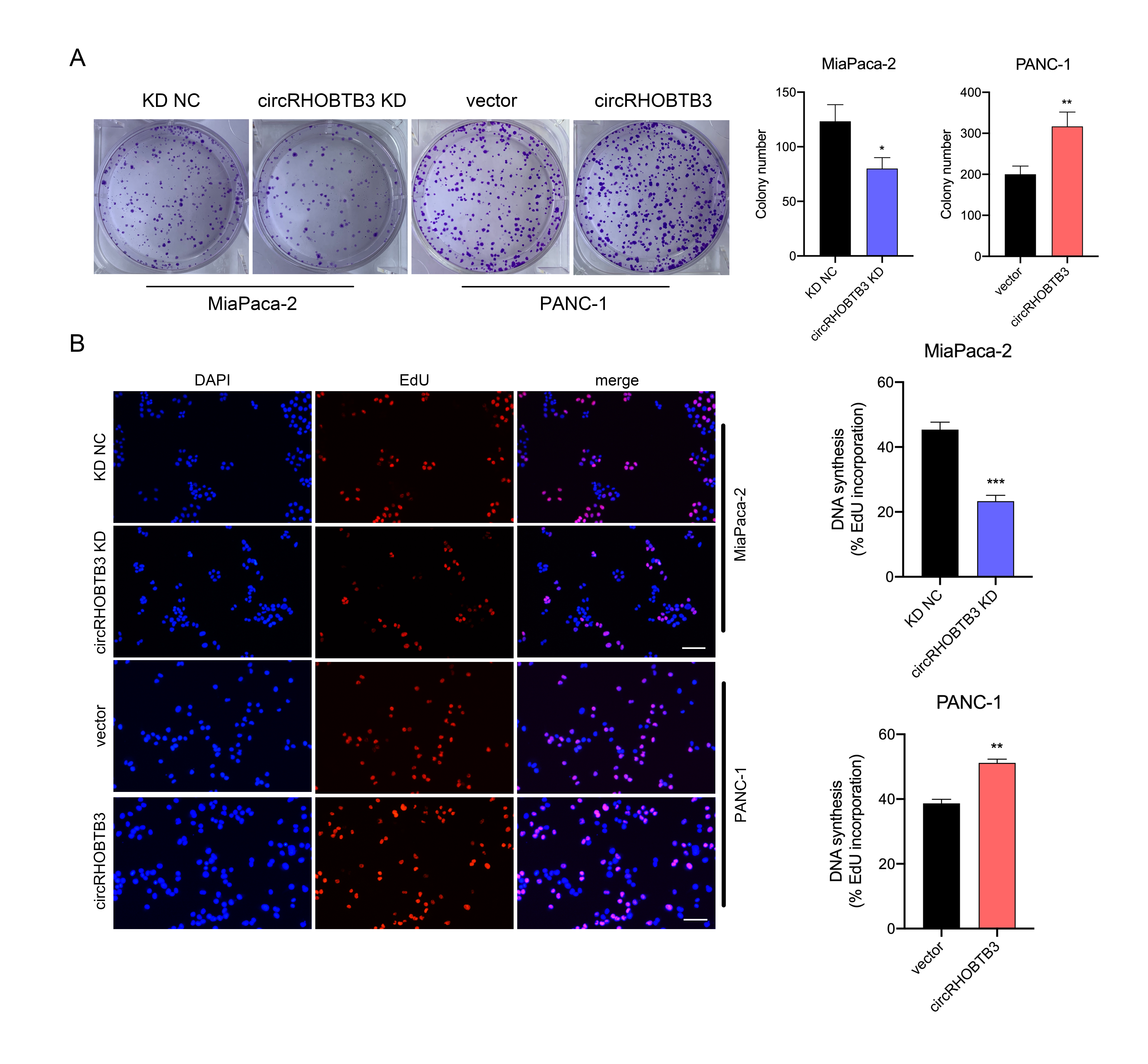

Supplement: Supplementary file 4 — Additional file 4: Figure S1. CircRHOBTB3 promotes PDAC cells proliferation in vitro. a. Colony formation assays showed that circRHOBTB3 facilitated PDAC cell proliferation. b. EdU assays in PANC-1 and MiaPaca-2 cells were performed to evaluate cell proliferation capabilities. The samples were imaged at 200× magnification. Scale bar = 50 μm. All data are presented as the means ± SD of three independent experiments. *p < 0.05, **p < 0.01, ***p < 0.001. [file 13046_2021_2063_MOESM4_ESM.jpg]

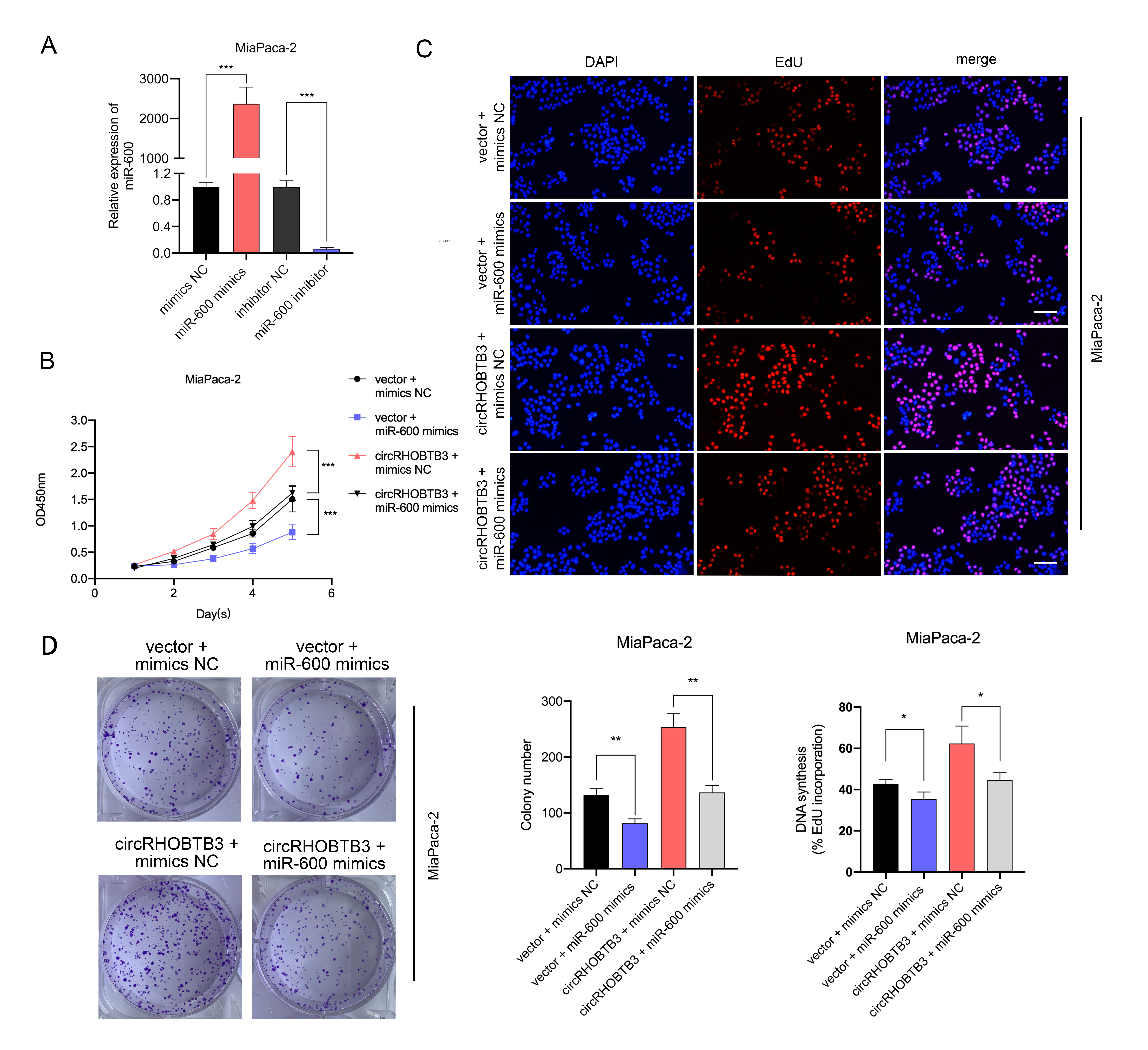

Supplement: Supplementary file 5 — Additional file 5: Figure S2. MiR-600 reverses the proliferation promotive effects by circRHOBTB3 overexpression in MiaPaca-2 cell line. a. The efficiency of miR-600 mimics and inhibitor were examined by qRT-PCR in MiaPaca-2 cell lines. b-d. Colony formation, EdU corporation assays and CCK-8 revealed that miR-600 abrogated the promoting role of circRHOBTB3 in MiaPaca-2 cell proliferation, The EdU samples were imaged at 200× magnification. Scale bar = 50 μm. All data are presented as the means ± SD of three independent experiments. *p < 0.05, **p < 0.01, ***p < 0.001. [file 13046_2021_2063_MOESM5_ESM.jpg]

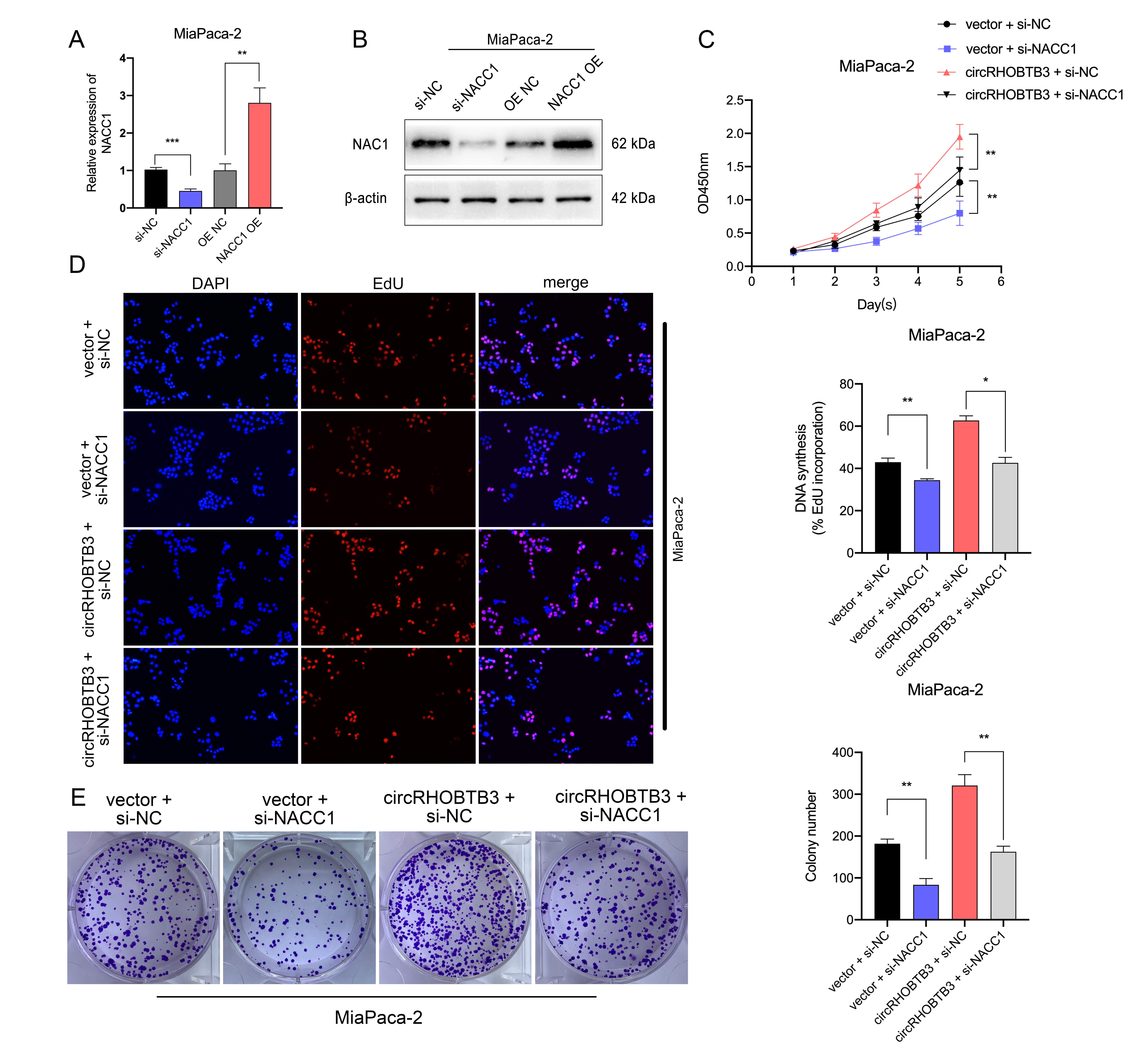

Supplement: Supplementary file 6 — Additional file 6: Figure S3. NACC1 knockdown reverses the oncogenic effect induced by circRHOBTB3 overexpression. a. The efficiency of NACC1 siRNA and overexpression vector is measured by qRT-PCR in MiaPaca-2. b. The protein levels of NACC1 knockdown and overexpression were determined by Western Blotting in MiaPaca-2 cell lines. c-e. MiaPaca-2 cells were divided into four groups (circRHOBTB3 vector + NACC1 si-NC, vector + NACC1 siRNA, circRHOBTB3 + NACC1 si-NC and circRHOBTB3 + NACC1 siRNA). The proliferation capabilities of MiaPaca-2 cells were detected through CCK-8, EdU corporation assays, colony formation assays. The EdU samples were imaged at 200× magnification. Scale bar = 50 μm. All data are presented as the means ± SD of three independent experiments. *p < 0.05, **p < 0.01, ***p < 0.001. [file 13046_2021_2063_MOESM6_ESM.jpg]
